# Physical and Functional Effects of Face-to-Face and Remotely Supervised Combined Exercise in Institutionalized Older Adults: A Non-Randomized Controlled Trial

**DOI:** 10.3390/healthcare14203539

**Published:** 2026-10-21

**Authors:** Ricardo Madeira, Dulce Esteves, Henrique P. Neiva, Nuno Pinto, Célia Nunes, Adriana Maia, Alessandro Vercelli, Maria Vaz Pato

**Affiliations:** 1 Department of Sport Sciences, University of Beira Interior, 6201-001 Covilhã, Portugal; desteves@ubi.pt (D.E.); hpn@ubi.pt (H.P.N.); adriana.maia1999@gmail.com (A.M.); 2 Research Centre in Sport Sciences, Health Sciences and Human Development (CIDESD), 6201-001 Covilhã, Portugal; 3 RISE-Health, University of Beira Interior, 6201-001 Covilhã, Portugal; nfcpinto@gmail.com (N.P.); mariavazpato@fcsaude.ubi.pt (M.V.P.); 4 Faculty of Health Sciences, University of Beira Interior, 6201-001 Covilhã, Portugal; 5 Department of Mathematics and Center of Mathematics and Applications, University of Beira Interior, 6201-001 Covilhã, Portugal; celian@ubi.pt; 6 Department of Neurosciences "Rita Levi Montalcini", University of Turin, 10126 Turin, Italy; alessandro.vercelli@unito.it

**Keywords:** aging, exercise, face-to-face, telemedicine, videoconference

## Abstract

**Highlights:**

**What are the main findings?**

- Significant Time × Group interactions were observed across body composition, strength, flexibility, functional mobility, and walking endurance outcomes (*p* < 0.001; ηp^2^ = 0.149–0.703).
- Both intervention groups improved across multiple outcomes over 12 weeks, whereas the control group remained stable or declined.
- No statistically significant differences in change were detected between the face-to-face and videoconference groups across most outcomes.

**What are the implications of the main findings?**

- Synchronous videoconference-supervised exercise may represent a promising delivery option when access to face-to-face exercise programmes in long-term care settings is limited.
- Combined exercise may support physical function in institutionalized older adults when delivered through either face-to-face or remotely supervised formats.

**Abstract:**

**Background/Objectives:** Supervised exercise is an important strategy for maintaining physical function in older adults; however, access to structured programmes may be limited in long-term care settings. Remotely supervised exercise may provide an alternative mode of delivery. This study examined the effects of videoconference-supervised and face-to-face combined exercise on physical and functional outcomes in institutionalized older adults. **Methods:** This three-arm non-randomized controlled trial included 87 institutionalized older adults assigned to a face-to-face exercise group, a videoconference-supervised exercise group, or a control group. Three participants did not complete follow-up, resulting in 84 participants with complete follow-up data who were included in the final analysis (mean age 83.13 ± 7.63 years). Both intervention groups completed the same 12-week combined exercise programme twice weekly, whereas the control group maintained usual activities. Body composition, muscular strength, flexibility, functional mobility, and aerobic endurance were assessed at baseline, 6 weeks, and 12 weeks. Time, Group, and Time × Group effects were examined using a mixed-design repeated-measures analysis. **Results:** Both exercise groups showed significant improvements in body composition, muscular strength, flexibility, functional mobility, and aerobic endurance, with significant Time × Group interactions and moderate-to-large effect sizes across these outcomes (*p* ≤ 0.001; ηp^2^ = 0.149–0.703). In the control group, body mass and BMI remained stable, whereas fat mass percentage, several strength outcomes, functional mobility, and aerobic endurance deteriorated over time. No statistically significant differences in change scores were observed between the face-to-face and videoconference groups (*p* > 0.05). **Conclusions:** Both supervised exercise formats were associated with favourable adaptations. Videoconference supervision may represent a viable delivery strategy when access to face-to-face programmes is limited. The study protocol was registered at ClinicalTrials.gov (registration number: NCT06722976; first posted on 9 December 2024).

## 1. Introduction

Population aging is one of the major global challenges of our time, resulting from advances in medical treatments and improved living conditions, which have significantly increased life expectancy worldwide [1,2,3]. This trend constitutes a global economic and social challenge, with direct implications for dependency and long-term care needs [4]. It is estimated that by 2050, the proportion of individuals aged 65 and older will reach 1.6 billion [5]. This growth in the percentage/absolute number of older adults brings critical implications for healthcare systems, with a substantial increase in the prevalence of non-communicable chronic diseases, such as diabetes, hypertension, cardiovascular diseases, dementia, and cancer [6,7,8]. Consequently, there is an urgent need to design and implement coordinated strategies aimed at fostering healthy and successful aging, thereby reducing the burden of chronic diseases and enhancing the quality of life in the elderly [9]. However, increases in longevity have not been accompanied by equivalent gains in healthy life expectancy, meaning that many older adults spend a substantial proportion of these additional years living with chronic diseases, disability, or reduced functional capacity. Therefore, the challenge is not only to extend lifespan but also to increase the number of years lived in good health, with preserved independence and quality of life [5].

Against this background, the concept of “successful aging” has emerged as a paradigm for improving the quality of life in older adults, prioritizing the maintenance of physical, cognitive, and social functionality, as well as reducing the period of morbidity before death [10,11,12]. Studies have highlighted that multiple factors influence successful aging, encompassing physiological, psychological, social, and lifestyle dimensions [13]. Within this framework, physical activity emerges as an essential tool, with robust evidence supporting its effectiveness in preventing chronic diseases, particularly cardiovascular disease and several types of cancer [14,15,16], increasing longevity and reducing all-cause mortality in older adults [16,17,18,19], improving cognitive function and attenuating age-related cognitive decline [20,21,22,23,24,25], and optimizing physical functionality, including strength, balance, mobility, and performance in activities of daily living [15,18,26,27,28,29]. Regular exercise contributes to cardiorespiratory fitness, muscular strength, and body composition, reducing the risk of dependency and promoting greater autonomy in old age [30]. In institutionalized older adults, maintaining physical fitness is essential for preserving independence and functional autonomy, as reductions in muscular strength, balance, and mobility are strongly associated with increased dependency in activities of daily living. Recent systematic reviews and meta-analyses specifically conducted in residential and long-term care settings indicate that structured exercise can improve physical performance, muscular strength, mobility, balance, and functional independence in institutionalized older adults [31,32]. Broader evidence synthesised from meta-analyses of older adults living in care settings also supports beneficial effects of physical activity across several physical and mental health outcomes, including mobility, functional dependency, and fall-related outcomes [33].

Despite the well-documented benefits of structured exercise, barriers such as cost, mobility limitations, and distance from training centres may restrict access to supervised programmes in older adults. In this context, digital technologies and remotely supervised exercise have gained increasing attention as alternative modes of delivery. Most of the available evidence on videoconference-delivered exercise, however, has been obtained in community-dwelling older adults. Recent reviews suggest that videoconference-based exercise can be feasible and may improve physical and functional outcomes in older populations, but these reviews largely excluded individuals living in residential or long-term care settings [34,35]. Similarly, direct comparisons between videoconference and face-to-face exercise have predominantly been conducted in healthy, community-dwelling older adults [36,37].

Consequently, evidence regarding videoconference-supervised exercise in institutionalized older adults remains limited. Residents of long-term care facilities may differ substantially from community-dwelling older adults in terms of age, frailty, multimorbidity, cognitive status, mobility limitations, and supervision requirements. These characteristics may influence the feasibility, safety, and physical response to remotely supervised exercise and limit the generalizability of findings obtained in community settings. This population gap provided the rationale for the present study. The present study therefore examined a videoconference-supervised exercise programme and a traditional face-to-face model, with particular emphasis on physical and functional outcomes in institutionalized older adults. Specifically, the study examined changes in upper- and lower-body strength, flexibility, mobility and agility, and aerobic endurance. The research question guiding this study is: Do changes in physical function differ between videoconference-supervised and face-to-face combined exercise programmes in institutionalised older adults?

## 2. Materials and Methods

### 2.1. Participants

**Figure 1:**
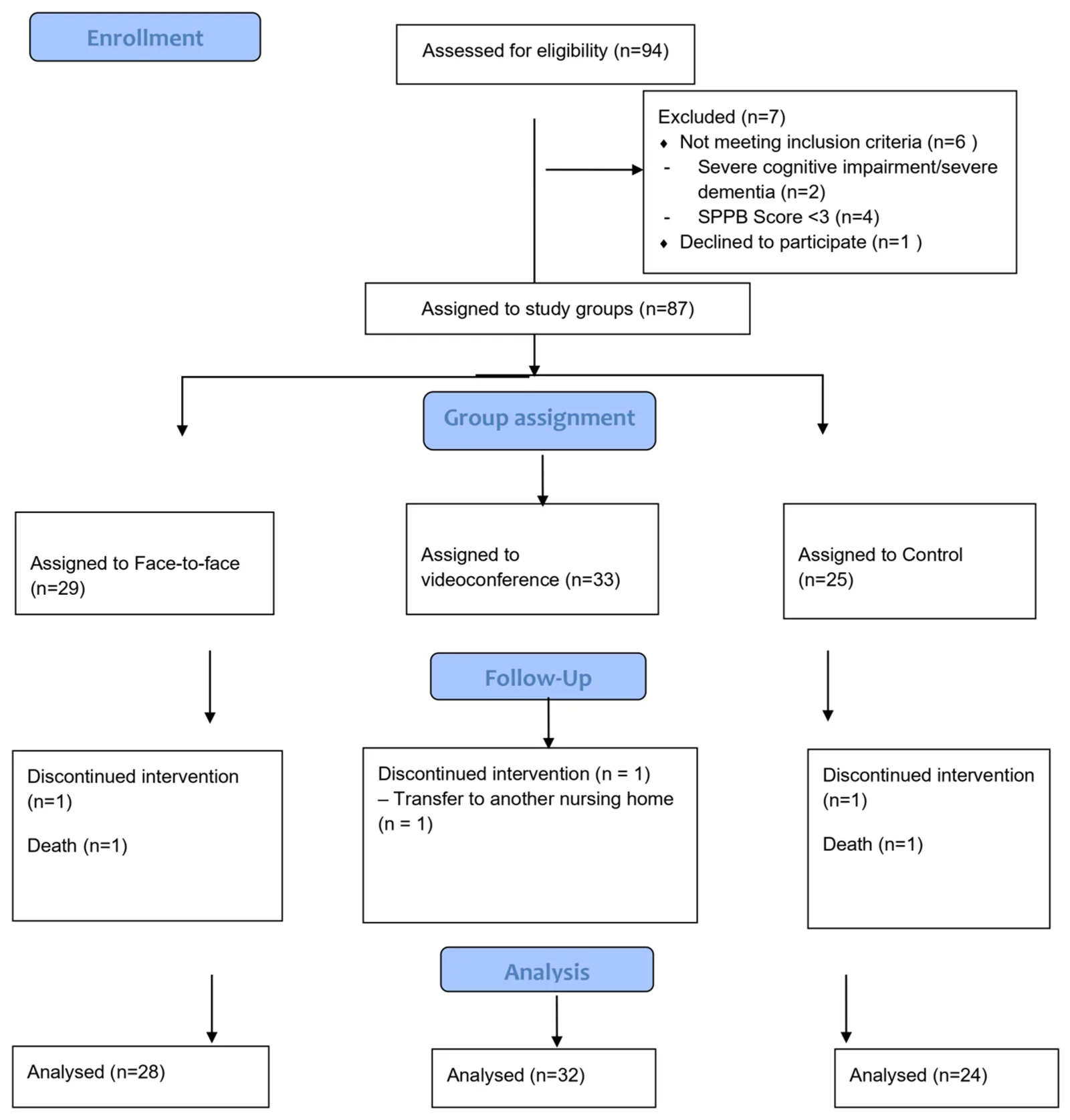
Flow diagram of participant recruitment, group assigned, follow-up, and analysis.

A total of 94 institutionalized older adults were assessed for eligibility. Seven individuals were excluded: six did not meet the eligibility criteria, including four with an SPPB score < 3 and two with severe cognitive impairment/severe dementia, and one declined to participate. Therefore, 87 participants were assigned to the three study groups: 29 to the face-to-face group (FG), 33 to the videoconference group (VG), and 25 to the control group (CG). Three participants did not complete follow-up: one participant in the FG died, one participant in the VG was transferred to another nursing home, and one participant in the CG died. Consequently, 84 participants were included in the final analysis (FG, *n* = 28; VG, *n* = 32; CG, *n* = 24). The participants were recruited from long-term care institutions located in the Beira Interior region of Portugal and had a mean age of 83.13 ± 7.63 years. Both intervention groups completed a 12-week combined exercise programme, whereas the control group maintained its usual activities without participating in a structured exercise programme. No serious intervention-related adverse events were reported during the study period. The recruitment, allocation, follow-up, and analysis of participants are presented in Figure 1. Recruitment was carried out across five nursing homes in the Beira Interior region that authorized participation. Clinical staff at each institution conducted initial health screenings to exclude participants who had uncontrolled hypertension or arrhythmia. All individuals were classified as sedentary, with no prior engagement in structured exercise programs during the three months preceding enrollment.

Potential participants were identified through collaboration between the principal investigator and the administrative and clinical staff of the participating institutions. All eligible individuals received detailed verbal and written information regarding the study procedures and voluntarily provided written informed consent prior to enrollment.

The study population comprised male and female participants aged 65 years or older who met the inclusion criteria of scoring ≥ 3 on the Short Physical Performance Battery (SPPB), demonstrating willingness to participate in the exercise interventions and collaborate with the research team, and possessing the ability to provide informed consent, either orally or in writing. Exclusion criteria included an SPPB score < 3, the presence of severe comorbidities that could compromise adherence or safety during the training program, severe dementia, or marked impairments across multiple cognitive domains that would hinder program participation. Additional exclusion criteria were recent surgical procedures or fractures within the preceding six months.

Cognitive status was initially screened using the Mini-Mental State Examination (MMSE), administered by trained psychologists, to help identify participants with severe cognitive impairment and support the exclusion of individuals with severe dementia. Information from participants’ clinical records and the clinical staff of the participating institutions was also considered when determining eligibility.

Prior to study initiation, all participants were informed about the objectives and procedures of the research and provided written informed consent. The experimental protocol adhered to the principles of the Declaration of Helsinki for research involving human subjects [38] and received formal approval from the Ethics Committee of the University of Beira Interior (protocol code CE-UBI-Pj-2023-064-ID1994; approved on 20 February 2024). The study is registered at ClinicalTrials.gov (registration no. NCT06722976; first posted on 9 December 2024).

### 2.2. Study Design

This investigation was conducted using a three-arm parallel non-randomized controlled design comparing face-to-face and videoconference-supervised exercise with a non-exercising control condition in institutionalized older adults.

After baseline assessments and confirmation of eligibility, participants were consecutively assigned within the participating institutions to one of three study groups: videoconference, face-to-face, or control. During the initial recruitment phase, group assignment followed the sequence videoconference–face-to-face–control. A minimum target of 25 participants had been established for the control group. Once this target was reached, no additional participants were assigned to the control condition, and subsequently enrolled participants were assigned between the videoconference and face-to-face intervention groups. This pragmatic assignment procedure resulted in unequal group sizes at the end of recruitment. Group assignment was therefore non-random, and no allocation concealment procedure was used.

Participants were allocated to one of three parallel groups: (a) a control group, which did not engage in structured exercise and continued with usual daily activities; (b) an intervention group that participated in a face-to-face physical training program; and (c) an intervention group that completed the same program delivered via videoconference. Both intervention groups followed an identical training protocol. Exercise sessions were conducted twice per week, with a 48 h recovery interval between sessions, and all interventions were scheduled in the morning. Participants assigned to the control group continued their usual daily routines throughout the 12-week study period and did not participate in any structured exercise programme. Usual activities consisted primarily of routine institutional activities and activities of daily living.

Although blinding of participants and exercise instructors was not feasible due to the nature of the intervention, outcome assessments and data analyses were performed by professionals who were not involved in group allocation and remained unaware of participants’ assigned conditions. Any withdrawals occurring during the study period were documented, including reasons when available.

The reporting of this non-randomized controlled trial followed the Transparent Reporting of Evaluations with Nonrandomized Designs (TREND) statement.

### 2.3. Classification of Medications Based on Cognitive and Functional Risk

Participants’ regular medication was analysed and classified according to its potential impact on cognition, balance, fall risk, and physical performance. This classification was based on the American Geriatrics Society Beers Criteria^®^ 2023, which identify potentially inappropriate medications in older adults and include drugs associated with adverse outcomes such as cognitive impairment, delirium, sedation, psychomotor impairment, orthostatic hypotension, falls, and fractures [39].

Dichotomous variables were created and coded as 0 = absence and 1 = presence to identify the use of medications with potential impact on: (a) cognitive performance, including anticholinergics, benzodiazepines, non-benzodiazepine hypnotics, antipsychotics, opioids, systemic corticosteroids, and H2-receptor antagonists; and (b) balance and physical performance, including anticholinergics, antidepressants, antiepileptics/gabapentinoids, antipsychotics, benzodiazepines, non-benzodiazepine hypnotics, opioids, skeletal muscle relaxants, medications associated with orthostatic hypotension/syncope, and medications with the potential to worsen motor symptoms.

### 2.4. Testing Procedures

All the measurements were taken in five different nursing homes. The tests were supervised and applied to all groups before, at 6 weeks and at 12 weeks in the exercise program (baseline, after 6 weeks and 12 weeks) by the same researchers.

All participants were assessed in person at their respective nursing homes by an experienced researcher involved in the study, along with three certified senior fitness trainers and five specialized psychologists who supervised the procedures to ensure safety and appropriate oversight during each evaluation. Assessments were conducted over three consecutive weeks with two weekly sessions, spaced 48 h apart for rest.

On the first day of physical assessments, participants completed the “6-min walk test, eight-foot up-and-go test, sit-and-reach test, and reach-behind-the-back test”. On the second day, the “sit-to-stand test, arm curl test, seated medicine ball throw, and handgrip strength tests” were administered. The third day was dedicated to body composition assessment. Psychological evaluations were conducted at baseline. At each assessment point, the psychological measures were administered over three consecutive days.

### 2.5. Measurements

#### 2.5.1. Physical Fitness Assessment

Physical fitness was assessed following the procedures of the Rikli and Jones test battery [40]. Six specific tests were carried out: the “6-min walk test” (6MWT), to assess aerobic endurance; the eight-foot up-and-go test (to assess agility and dynamic balance); the sit-stand test (to measure lower limb strength); the arm curl test (to measure upper limb strength); the sitting and reaching test; and the reach behind the back test (to assess lower/upper body flexibility). The seated medicine ball throw and handgrip strength tests were also added to the physical fitness assessment. The assessment procedure for each of the tests is detailed in the following points.

#### 2.5.2. Body Composition

The percentage of fat mass was assessed using bioelectrical impedance analysis (BC-613; TANITA, Tokyo, Japan), following standardized pre-assessment conditions (e.g., fasting state, avoidance of exercise and alcohol, controlled hydration status) as recommended for bioelectrical impedance measurements in older adults [41]. Body fat percentage and fat-free mass estimates were obtained using the prediction algorithm incorporated into the device; no separate prediction equation was applied by the research team. Assessments were performed under standardized pre-assessment conditions, including control of recent food intake, exercise, alcohol consumption, and hydration status, as far as practically feasible within the institutional setting. Body mass index (BMI) was calculated as weight (kg) divided by height squared (m^2^). Height measurements were taken using a stadiometer (Cabral, model 14, Balanças Cabral – António Antunes & Filhos, Lda., Braga, Portugal). Participants removed their shoes and stood upright with feet flat on the floor, heels in contact, and their trunk and head positioned together against the stadiometer. The head position was neutral with the chin neither lifted nor tucked. Height measurements were recorded after participants exhaled.

#### 2.5.3. Sit-Stand Test

The sit-stand test was employed to indirectly assess lower limb muscle strength and functionality. Participants used a chair with a 40 cm height and a straight backrest [42]. With arms folded across the chest and plantar support on the floor throughout, participants were instructed to stand up and sit down as many times as possible within 30 s [42]. The outcome of this test was determined by counting the number of times the participant successfully completed the sit-stand cycle [42].

#### 2.5.4. Seated Medicine Ball Throw Test

The seated medicine ball throw test measured upper body strength and power. Participants were seated on a chair, with the trunk against the chair back, holding a 3 kg medicine ball in front of the trunk with both hands. Rotation of the trunk and hips was restricted during testing [42]. Three throws were performed with one-minute rest intervals to prevent fatigue [42]. The distance from the point of release to where the ball contacted the ground was measured using a metric tape. The mean distance of the throws was used for subsequent analysis [42].

#### 2.5.5. Handgrip Strength Test

Handgrip strength test, indicative of upper limb muscle function, was measured using an adjustable digital hand dynamometer (Saehan, Model DHD-1, Changwon, Republic of Korea). Participants were seated with hips, knees, and elbows flexed at 90°, gripping the dynamometer in a neutral forearm position [43]. Three measurements of grip strength were taken for each hand, with a two-minute rest period between attempts. The average of these measurements was used for data analysis.

#### 2.5.6. Arm Curl Test

In the arm curl test, participants sat on a chair and were asked to flex the elbow of the strongest arm as many times as possible in 30 s while holding a 3 kg dumbbell [44]. Participants were instructed to maintain a normal breathing pattern and keep the elbow at the side of the body throughout the test [44]. A single repetition consisted of complete elbow flexion from maximum extension [44].

#### 2.5.7. Back Scratch Test

While standing, participants are to position their dominant hand over their shoulder, reaching as far down the middle of their back as possible. The palm should face downward and the fingers must be extended (with the elbow pointing towards the ceiling) [40]. The other hand is then placed underneath and behind the back, with the palm facing upward, and should reach up as far as possible. The objective is to touch or overlap the middle fingers of the two hands.

#### 2.5.8. Chair Sit-and-Reach Test

To begin, participants will be seated and move forward until they are sitting on the edge of the chair, making sure the crease where the upper thigh meets the buttocks is even with the front edge of the seat. One leg must remain bent with the foot flat on the floor, while the preferred leg is extended straight ahead, with the heel resting on the floor and the foot bent at roughly a 90° angle [40].

Participants should be told to exhale as they gently lean forward, being careful to avoid any sudden or forceful motions, and to stop immediately if they feel any pain. While keeping the extended leg straight (avoiding hyperextension), they must slowly bend forward at the hip joint, maintaining a straight spine and keeping the head aligned with the spine to prevent the neck from bending. With the hands stacked one on top of the other and the fingertips overlapping, the participants will slide their hands down the extended leg to reach the toes.

#### 2.5.9. Eight-Foot up and Go Test

A chair was placed at the starting point with a cone positioned 8 feet (2.44 m) away [45]. Participants, starting seated with hands on knees, stood up, walked quickly around the cone, and returned to the chair, with timing starting on “go” and stopping when they sat back down [45].

#### 2.5.10. 6-Min Walk Test

At the start signal, the subjects were instructed to walk as fast as possible (without running) for 6 min [42]. During the test, the supervisor walked slightly behind the participant, avoiding accompanying them side by side so as not to influence the self-selected walking pace [42].

### 2.6. Intervention: Training Program

The face-to-face and videoconference groups carried out the same training program over 12 weeks, with two weekly 1 h sessions. All exercise sessions were supervised by the same qualified exercise professional specialized in exercise prescription. The face-to-face and videoconference sessions were scheduled separately and were not delivered simultaneously. Using the same instructor across both intervention groups was intended to standardize exercise instruction, progression, and supervision between delivery formats. The programme consisted of a combined exercise intervention integrating aerobic capacity and muscle strength of older adults. Each training session included a 5–10 min warm-up, 35–40 min of combined exercise and a 5–10 min cool-down. Training intensity was assessed using a pulse cardiofrequency meter (Polar unite, 2020, Kempele, Finland). Maximum heart rate was estimated individually using the age-predicted equation HRmax = 208 − (0.7 × age), and the target training intensity was calculated as a percentage of each participant’s estimated HRmax. The intensity was prescribed at between 50% and 70% of the maximum heart rate [26,46,47]. From weeks 1 to 4, the intensity was set between 50 and 55% HRmax; in weeks 5–8 it was set between 55 and 60% HRmax; and in weeks 9–12 it increased to 60 to 70% HRmax. Heart rate was monitored in real time during exercise sessions using Polar Unite devices to support adherence to the prescribed intensity ranges. However, session-level heart-rate data were not systematically stored for subsequent analysis. Regarding resistance training, the sessions typically included 1 to 4 sets [20,26]. The groups performed the same volume (sets × repetitions) (week 1, 1 × 10; weeks 2–3, 2 × 10; weeks 4–5, 3 × 10; weeks 6–9, 4 × 8; weeks 10–12, 4 × 10) [48]. The detailed training program is described in more detail in the Supplementary Table S1.

Videoconference sessions were delivered synchronously using Zoom Video Communications Inc. (San Jose, CA, USA), 2016. The exercise instructor supervised participants remotely in real time using camera/audio and a pulse cardiofrequency meter, which enabled continuous visual and verbal interaction throughout the sessions. Approximately 5 participants took part in each videoconference session. A trained staff member remained physically present at the institution during all remote sessions and was responsible for technical setup, management of connectivity or audiovisual issues, participant positioning, and safety support, as applicable. Exercise prescription, demonstration, progression, and correction remained the responsibility of the exercise instructor.

### 2.7. Safety Monitoring

To ensure participant safety during videoconference sessions, a trained technician remained present throughout each session to address potential technical issues and respond to any adverse events. All incidents were systematically recorded and categorized as either technical (e.g., connectivity disruptions) or participant-related (e.g., pain, discomfort, or health-related symptoms). Events were further classified according to severity as minor (allowing continuation of the session) or major (requiring interruption of the session).

In cases where participants reported pain that worsened during training, exercise modification followed a structured four-phase model [49]. The first step consisted of reducing the load by decreasing the resistance or weight used during the exercise. In some situations, this reduction could reach 100%, meaning that the movement was performed without any external resistance. If pain persisted, the second step involved slowing down the movement execution to minimize mechanical strain. When necessary, the third step was to reduce the range of motion (ROM), limiting the movement to a pain-free range while maintaining sufficient amplitude to ensure effective dynamic strength training. If none of these adjustments alleviated the pain, the final step was to discontinue the exercise.

### 2.8. Sample Size Calculation

An a priori power analysis was performed using G*Power (version 3.1.9.7; Heinrich Heine University Düsseldorf, Düsseldorf, Germany) to determine the required sample size to detect the primary effect of interest, i.e., the Time × Group interaction, in a mixed-design model with three groups (face-to-face, videoconference, control) and three measurements (baseline, 6 weeks, 12 weeks). The analysis assumed a significance level of α = 0.05, power (1 − β) = 0.80, a correlation among repeated measures of r = 0.50, and a nonsphericity correction ε = 1.0. A medium effect size was specified (a Cohen’s f = 0.25, equivalent to ηp^2^ ≈ 0.06). Under these assumptions, the minimum required sample size was estimated at 36 participants (12 per group). To account for a potential attrition rate of approximately 15%, the target sample size was increased to 42 participants (14 per group). The final sample included 84 participants, exceeding the minimum sample size required to achieve adequate statistical power.

### 2.9. Statistical Analysis

Data analysis was performed using Microsoft Excel 2013 (Microsoft Corporation, Redmond, WA, USA) and IBM SPSS Statistics for Windows, version 28.0 (IBM Corp., Armonk, NY, USA). Descriptive statistics were computed for all variables and are presented as means and standard deviations. Normality was assessed using the Kolmogorov–Smirnov test [50] and homogeneity of variances was evaluated using Levene’s test. Sphericity was assessed using Mauchly’s test. When the assumption of sphericity was violated, Greenhouse–Geisser-corrected degrees of freedom and *p*-values were used.

To examine the effects of the intervention over time and between groups, a two-way mixed-design ANOVA was conducted, with Time (baseline, 6 weeks, and 12 weeks) as the within-subject factor and Group (face-to-face group, videoconference group, and control group) as the between-subject factor. For all outcome variables, the Time × Group interaction was analyzed, and the main effects of Time and Group were evaluated when appropriate. When significant Time × Group interaction or main effect was detected, pairwise comparisons were conducted, with Bonferroni correction applied to adjust for multiple comparisons. Effect sizes were expressed as partial eta squared (ηp^2^) and interpreted according to established thresholds: small (ηp^2^ ≥ 0.01), medium (ηp^2^ ≥ 0.06), and large (ηp^2^ ≥ 0.14) [51].

Given the observed baseline differences in age and sex distribution between groups, an adjusted sensitivity analysis was additionally performed using repeated-measures analysis of covariance (ANCOVA). Age and BMI were included as covariates and sex as an additional between-subject factor, while Time (baseline, 6 weeks, and 12 weeks) and Group remained the within- and between-subject factors, respectively. The adjusted Time × Group interaction was examined for each outcome to determine whether the longitudinal pattern of results remained consistent after accounting for age and sex. Effect sizes were expressed as partial eta squared (ηp^2^). To further assess the potential influence of medication-related confounding, an additional sensitivity analysis was conducted among participants with available medication data. Age, baseline BMI, and the number of CNS-active medications were included as covariates, while sex was included as an additional between-subject factor. Time and Group remained the within- and between-subject factors, respectively. The adjusted Time × Group interaction was examined for all non-BMI outcomes.

To further evaluate whether the groups exhibited distinct trajectories of change over time, an additional analysis based on differences from baseline (Δ) was conducted. For each variable, Δ values were calculated as the differences between baseline and 6 weeks (Δ0–6) and between baseline and 12 weeks (Δ0–12). Between-group comparisons of the Δ values were performed using the nonparametric Kruskal–Wallis test, due to violations of normality, followed by pairwise comparisons with Bonferroni correction. This approach allowed direct assessment of the relative superiority of the interventions compared with the control condition and enabled comparison of the effectiveness of the two supervision formats over time. Statistical significance was set at *p* ≤ 0.05 for all analyses.

## 3. Results

Baseline characteristics of the participants are presented in Table 1, including demographic and anthropometric variables, cognitive status, medication-related variables, and prior physical activity across the face-to-face, videoconference, and control groups. Of the 87 participants assigned to the study groups (FG, *n* = 29; VG, *n* = 33; CG, *n* = 25), three did not complete follow-up: one participant in the FG died, one participant in the VG was transferred to another nursing home, and one participant in the CG died. Consequently, 84 participants were included in the final analysis (FG, *n* = 28; VG, *n* = 32; CG, *n* = 24) (Figure 1).

Table 2 summarizes the longitudinal effects of a 12-week face-to-face and videoconference-supervised combined exercise program on body composition, physical fitness, and functional outcomes in institutionalized older adults. Mean ± SD values are presented for baseline, 6 weeks, and 12 weeks for face-to-face, videoconference, and control groups. Additionally, the table reports the results of the two-way mixed-design ANOVA, including main effects of time, group, and the time × group interaction. A total of 84 participants completed the physical fitness assessments at all three time points (baseline, 6 weeks, and 12 weeks) and were included in the final analysis. The face-to-face group comprised 28 participants (25 women and 3 men), with a mean age of 85.75 ± 7.58 years and a mean height of 1.54 ± 0.09 m. The videoconference group comprised 32 participants (20 women and 12 men), with a mean age of 80.06 ± 6.82 years and a mean height of 1.58 ± 0.09 m. The control group comprised 24 participants (15 women and 9 men), with a mean age of 84.17 ± 7.63 years and a mean height of 1.66 ± 0.05 m.

Table 3 summarizes the results of the post hoc multiple comparisons, detailing within-group changes observed between assessment periods (baseline–6 weeks, baseline–12 weeks, and 6–12 weeks) for all outcome variables, allowing characterization of the temporal pattern and magnitude of adaptations within each group.

To further examine whether the intervention groups differed in their trajectories of change over time, an additional between-group analysis was performed based on the differences from the baseline (Δ) (between baseline and 6 weeks and between baseline and 12 weeks). This analysis allowed direct comparison of the magnitude of change between the Face-to-Face, Videoconference, and Control groups. The results of these independent nonparametric comparisons are presented in Supplementary Table S2, providing complementary evidence to the main time × group interaction effects reported in the primary analyses.

At baseline, no statistically significant differences were observed between groups regarding medication-related variables, including the number of medications with cognitive impact, physical/balance impact, combined physical/balance/cognitive impact, active medications in the central nervous system, or the use of three or more central nervous system-active medications (Supplementary Table S3). This pattern was consistent across the total baseline sample, with all pairwise comparisons between the Face-to-Face, Videoconference and Control Group showing non-significant *p*-values.

### 3.1. Body Composition and Anthropometric Variables

For body weight, no significant Time × Group interaction was observed. In the absence of interaction, no significant main effects of Time or Group were detected, indicating stability of body weight across all groups and assessment moments.

Regarding body mass index (BMI), no significant Time × Group interaction was found. A significant main effect of Group was observed (F = 6.928; *p* = 0.002; ηp^2^ = 0.146), indicating consistent differences between groups over time, while no significant effect of Time was detected.

For fat mass percentage, a significant Time × Group interaction was observed (F = 94.475; *p* < 0.001; ηp^2^ = 0.703). Post-hoc analyses (Table 3) revealed significant reductions in the face-to-face and videoconference groups across all time intervals (baseline–6 weeks, baseline–12 weeks, and 6–12 weeks; *p* < 0.001), whereas the control group showed no improvement and a significant increase between weeks 6 and 12 (*p* = 0.018).

Similarly, fat-free mass showed a significant Time × Group interaction (F = 29.789; *p* < 0.001; ηp^2^ = 0.436). Post-hoc analyses (Table 3) indicated significant increases in fat-free mass in both intervention groups at all time intervals (*p* < 0.001), while no significant changes were observed in the control group.

For waist circumference, a significant Time × Group interaction was identified (F = 17.846; *p* < 0.001; ηp^2^ = 0.311). Both intervention groups exhibited significant reductions at all assessment intervals (*p* < 0.001), whereas no significant changes were observed in the control group (Table 3).

Comparable results were observed for hip circumference, with a significant Time × Group interaction (F = 29.284; *p* < 0.001; ηp^2^ = 0.429). Significant reductions occurred in both intervention groups across all time intervals (*p* < 0.001), while the control group showed no significant changes (Table 3).

Consistent with these findings, the between-group analysis based on the differences from baseline (Δ) indicated that both FG and VG showed significant reductions in fat mass percentage, waist circumference, and hip circumference, as well as greater increases in fat-free mass, compared with the control group at both baseline–6 weeks and baseline–12 weeks (FG vs. CG and VG vs. CG: *p* < 0.001). No statistically significant differences in Δ0–6 or Δ0–12 were detected between the face-to-face and videoconference groups for any body composition or anthropometric outcome (*p* > 0.05; see Supplementary Table S2).

### 3.2. Functional Fitness Outcomes

For the sit-to-stand test, a significant Time × Group interaction was observed (F = 27.288; *p* = 0.001; ηp^2^ = 0.403), indicating different performance trajectories across groups over time. Post-hoc analyses (Table 3) showed significant improvements in both the face-to-face and videoconference groups at all time intervals (*p* < 0.01), whereas no significant changes were observed in the control group.

For the seated medicine ball throw, a significant Time × Group interaction was also identified (F = 37.201; *p* < 0.001; ηp^2^ = 0.479). Both intervention groups demonstrated significant increases across all assessment intervals (*p* < 0.001), while the control group showed a significant decline between weeks 6 and 12 and between baseline and week 12 (Table 3).

Similarly, handgrip strength of the right hand revealed a significant Time × Group interaction (F = 54.345; *p* < 0.001; ηp^2^ = 0.573). Post-hoc analyses (Table 3) indicated significant improvements in both intervention groups at all time intervals (*p* < 0.001), whereas the control group experienced significant declines over time.

Handgrip strength of the left hand also showed a significant Time × Group interaction (F = 51.007; *p* < 0.001; ηp^2^ = 0.557). Significant increases were observed in the face-to-face and videoconference groups across all time intervals (*p* < 0.001), while the control group exhibited a significant reduction between baseline and week 12 (Table 3).

For the arm curl test, a significant Time × Group interaction was observed (F = 27.700; *p* < 0.001; ηp^2^ = 0.406), together with significant main effects of Time (F = 65.260; *p* < 0.001; ηp^2^ = 0.446) and Group (F = 3.884; *p* = 0.025; ηp^2^ = 0.088) (Table 1). Post hoc analyses (Table 2) showed significant improvements in both the face-to-face and videoconference groups across all assessment intervals (baseline–6 weeks, baseline–12 weeks, and 6–12 weeks; *p* < 0.001), whereas no statistically significant changes were observed in the control group (*p* ≥ 0.128).

Consistent with these findings, the between-group analysis based on the differences from baseline (Δ) demonstrated that both FG and VG achieved significantly greater improvements in lower- and upper-limb strength compared with the control group at both baseline–6 weeks and baseline–12 weeks (FG vs. CG and VG vs. CG: *p* < 0.001). No statistically significant differences in change were detected between the face-to-face and videoconference groups for any strength-related outcome (*p* ≥ 0.336; see Supplementary Table S2).

### 3.3. Upper and Lower—Flexibility

For the back scratch test (right), a significant Time × Group interaction was observed (F = 34.597; *p* < 0.001; ηp^2^ = 0.467), indicating different flexibility adaptations across groups over time. Post-hoc analyses (Table 3) showed significant improvements in both the face-to-face and videoconference groups across all time intervals, whereas no significant changes were observed in the control group.

Comparable results were found for the back scratch test (left), with a significant Time × Group interaction (*p* < 0.001). Improvements were observed exclusively in the face-to-face and videoconference groups, with no significant changes in the control group (Table 3).

For the chair sit-and-reach test (right leg), a significant Time × Group interaction was identified (F = 7.100; *p* < 0.001; ηp^2^ = 0.149). Both intervention groups demonstrated significant improvements across all assessment intervals, while no significant changes were observed in the control group (Table 3).

Similarly, the chair sit-and-reach test (left leg) showed a significant Time × Group interaction (*p* < 0.001). Significant improvements were observed in both intervention groups, whereas the control group showed no significant changes (Table 3).

Consistent with these findings, the between-group analysis based on the differences from baseline (Δ) generally indicated more favourable changes in upper- and lower-limb flexibility in the intervention groups compared with the control group at both baseline–6 weeks and baseline–12 weeks. However, for the left chair sit-and-reach test, the difference between the videoconference and control groups from baseline to 12 weeks did not reach statistical significance (*p* = 0.064). No significant differences were observed between the Face-to-Face and Videoconference groups for any flexibility outcome (*p* ≥ 0.065) (see Supplementary Table S2).”

### 3.4. Agility, Dynamic Balance, and Cardiorespiratory Fitness

For the 8-foot up-and-go test, a significant Time × Group interaction was observed (F = 48.574; *p* < 0.001; ηp^2^ = 0.552), indicating different agility trajectories across groups over time. Post-hoc analyses (Table 3) showed significant reductions in completion time in both the face-to-face and videoconference groups at all assessment intervals, whereas the control group exhibited significant worsening over time.

For the 6-min walk test, a significant Time × Group interaction was also identified (F = 7.664; *p* < 0.001; ηp^2^ = 0.168). Post-hoc analyses (Table 3) showed that the face-to-face group did not demonstrate a statistically significant improvement from baseline to 6 weeks (*p* = 0.423), but significant improvements were observed from baseline to 12 weeks (*p* = 0.017) and from 6 to 12 weeks (*p* = 0.002). In contrast, the videoconference group showed significant improvements across all assessment intervals, while the control group showed significant declines from baseline to 6 weeks and from baseline to 12 weeks.

Consistent with these findings, the between-group analysis based on the differences from baseline (Δ) demonstrated that both FG and VG achieved significantly greater improvements than the control group in the 8-foot up-and-go test at both baseline–6 weeks and baseline–12 weeks (FG vs. CG and VG vs. CG: *p* < 0.001). For the 6-min walk test, both intervention groups showed a significantly more favorable evolution than CG at baseline–12 weeks (FG vs. CG: *p* = 0.012; VG vs. CG: *p* < 0.001), with no significant differences observed between the Face-to-Face and Videoconference groups for either outcome (*p* ≥ 0.270; see Supplementary Table S2).

### 3.5. Sensitivity Analyses Adjusted for Age, Sex, Baseline BMI and CNS-Active Medication Burden

Adjusted sensitivity analyses controlling for age and sex yielded results that were consistent with the primary analyses. To further account for the observed baseline differences in BMI between groups, a second sensitivity analysis was performed for all non-BMI outcomes, with age and baseline BMI included as covariates and sex included as an additional between-subject factor. The overall pattern of findings remained consistent after this additional adjustment. The Time × Group interaction remained non-significant for body mass (F = 1.487, *p* = 0.214, ηp^2^ = 0.038), whereas significant Time × Group interactions were retained for body fat percentage, fat-free mass, waist circumference, hip circumference, sit-to-stand performance, seated medicine ball throw, right- and left-handgrip strength, arm curl, right- and left-back scratch, right- and left-chair sit-and-reach, 8-foot up-and-go, and 6-min walk test (all *p* ≤ 0.008). Full results of the analyses additionally adjusted for baseline BMI, including F statistics, *p*-values, and partial eta squared (ηp^2^), are presented in Supplementary Table S4.

To further examine the potential influence of medication-related confounding, an additional sensitivity analysis was conducted. In this model, age, baseline BMI, and the number of CNS-active medications were included as covariates, while sex was retained as an additional between-subject factor. The overall pattern of findings remained consistent after this additional adjustment. The Time × Group interaction remained non-significant for body mass (F = 1.290, *p* = 0.279, ηp^2^ = 0.036), whereas significant Time × Group interactions were retained for body fat percentage, fat-free mass, waist circumference, hip circumference, sit-to-stand performance, seated medicine ball throw, right- and left-handgrip strength, arm curl, right- and left-back scratch, right- and left-chair sit-and-reach, 8-foot up-and-go, and 6-min walk test (all *p* ≤ 0.018). BMI was not included as an outcome in analyses incorporating baseline BMI as a covariate. Full results of the sensitivity analyses, including models adjusted for age and sex, baseline BMI, and CNS-active medication burden, including F statistics, *p*-values, and partial eta squared (ηp^2^), are presented in Supplementary Table S4.

### 3.6. Adherence, Technical Events, and Safety

Session attendance was 92% in FG and 90% in VG across the 24 scheduled sessions. Five technical disruptions were recorded during the videoconference intervention, mainly involving temporary internet connection problems, audio/video interruptions, or equipment-related issues. No serious intervention-related adverse events occurred. No falls or cardiovascular events related to the intervention were recorded. Minor musculoskeletal discomfort was reported by 6 of the 62 participants in the intervention groups (9.7%), including 3 participants in the face-to-face group and 3 in the videoconference group. These events were managed according to the predefined four-stage pain-monitoring and exercise-modification procedure, with exercise load, movement speed, range of motion, or the individual exercise modified when necessary.

## 4. Discussion

The present non-randomized controlled study examined changes in body composition, muscular strength, flexibility, functional mobility, and aerobic endurance associated with face-to-face and videoconference-supervised combined exercise in institutionalized older adults over 12 weeks. Both supervised exercise groups showed favorable changes across most outcomes, whereas the control group showed no improvement or deterioration in several variables. Significant Time × Group interactions were observed for most outcomes, indicating different trajectories of change across the three groups. Overall, both supervised exercise modalities were associated with improvements across the majority of physical and functional outcomes assessed. No statistically significant differences in change were detected between the face-to-face and videoconference groups across most outcomes.

### 4.1. Body Composition

Both intervention groups demonstrated significant improvements in body composition, characterized by reductions in fat mass percentage, waist circumference, and hip circumference, along with increases in fat-free mass at both 6 and 12 weeks. These changes were accompanied by large effect sizes and significant time × group interactions, indicating distinct trajectories between the intervention groups and the control group. In contrast, CG showed no beneficial changes, and in some variables, such as fat mass percentage, exhibited a worsening over time.

The absence of significant changes in body weight suggests that the favorable adaptations were driven by qualitative changes in body composition rather than by overall mass reduction, which is consistent with the known effects of combined exercise in older adults. No statistically significant differences in change were detected between FG and VG for the body-composition outcomes. However, this absence of statistically significant differences should not be interpreted as evidence of equivalent effectiveness between the two delivery formats. These findings are consistent with previous evidence showing that combined aerobic and resistance training effectively improves adiposity-related outcomes in older adults, regardless of delivery format, when exercise content and supervision are maintained [36,37,52,53].

Importantly, potential confounding factors were not fully controlled and should be considered when interpreting these findings. These include dietary habits, medication use (e.g., corticosteroids or other drugs that may influence muscle mass, inflammation, or fluid retention), and other lifestyle-related variables that could have affected body composition outcomes. Therefore, although the results suggest a positive effect of the intervention, caution is warranted when attributing the observed changes exclusively to the exercise program.

### 4.2. Muscular Strength

Significant improvements were observed in all strength-related outcomes, including the sit-to-stand test, seated medicine ball throw, handgrip strength (right and left hand), and arm curl test, in both FG and VG. The presence of significant time, group, and time × group effects across these measures indicates different trajectories of change across groups, with favorable improvements observed in both intervention groups.

Notably, improvements occurred early (within the first 6 weeks) and were maintained or further enhanced by week 12 in both intervention groups. The control group either showed no change or experienced declines, particularly in handgrip strength, whereas favorable changes were observed in both supervised exercise groups.

Crucially, no consistent or systematic differences between FG and VG were detected in the strength outcomes based on the current analyses. No statistically significant differences in change were detected between the two supervised modalities for the strength-related outcomes. These findings are broadly consistent with previous studies reporting favorable strength-related adaptations following remote and in-person supervised exercise in older adults [36,54,55]. However, most of these studies were conducted in healthy or community-dwelling older adults rather than in institutionalized populations, and direct comparisons should therefore be interpreted cautiously.

### 4.3. Flexibility

Flexibility, assessed through the back scratch test (right and left) and the chair sit-and-reach test (right and left), improved significantly in both intervention groups across all time points. Significant time × group interactions indicated different trajectories of change across groups, with improvements observed in both intervention groups while the control group showed no significant changes.

Both FG and VG exhibited early gains in 6 weeks that were sustained or augmented at 12 weeks, indicating that flexibility adaptations occurred rapidly and continued with ongoing training. Importantly, the current results do not demonstrate consistent statistical superiority of one intervention modality over the other. Improvements were evident in both groups for both upper and lower flexibility, with no consistent evidence of statistical superiority of one supervised delivery format over the other.

These findings are broadly consistent with previous literature reporting flexibility improvements following in-person and remotely supervised exercise in older adults [36,55,56,57]. Nevertheless, much of this evidence derives from community-dwelling or otherwise healthier older populations, which may differ substantially from nursing-home residents in functional status and supervision requirements.

### 4.4. Functional Mobility and Aerobic Endurance

Both FG and VG showed significant improvements in functional mobility, as assessed by the 8-foot up-and-go test, with reductions in completion time observed at 6 and 12 weeks. In contrast, CG exhibited significant worsening over time, highlighting the negative impact of inactivity on agility and dynamic balance in institutionalized older adults.

Similarly, aerobic endurance, measured by the 6-min walk test, improved significantly in both intervention groups, with significant time × group interactions. While the temporal pattern of improvement differed slightly, with VG showing significant gains earlier and FG reaching significance by 12 weeks, the control group demonstrated a decline in walking distance. From a clinical perspective, the magnitude of the improvements observed at 12 weeks may also be meaningful. Changes of approximately 20 m and 50 m in 6-min walk distance may represent small and substantial meaningful changes, respectively, in older adults [58], while a minimal clinically important difference of 17.8 m in moderately frail older adults. In the present study, walking distance increased by 40.5 m in FG and 68.8 m in VG from baseline to 12 weeks, exceeding these published thresholds [59]. In the present study, walking distance increased by 40.5 m in FG and 68.8 m in VG from baseline to 12 weeks, exceeding these published thresholds. However, these reference values were derived from populations that were not identical to institutionalized older adults and should therefore be interpreted cautiously.

No statistically significant differences in change were detected between FG and VG in agility and aerobic endurance outcomes. These findings suggest that videoconference-supervised exercise may represent a promising delivery option when face-to-face supervision is difficult to provide; however, they should not be interpreted as evidence of equivalent effectiveness between the two delivery formats. These findings are broadly consistent with previous studies reporting favorable functional changes following both remote and in-person exercise interventions [36,54,55]. However, these studies predominantly involved healthy or community-dwelling older adults. The present study therefore extends this evidence by examining videoconference-supervised exercise in institutionalized older adults, a population that has been underrepresented in previous remote-exercise research.

This population typically presents advanced age, reduced physiological reserve, high prevalence of chronic disease, sarcopenia, and elevated levels of physical inactivity [60,61,62]. In many nursing homes, structured exercise programs are minimal or nonexistent, and daily routines are predominantly sedentary, with residents spending a substantial proportion of waking hours inactive [63,64]. Therefore, the magnitude of improvements observed in the intervention groups may reflect not only the effectiveness of the training model itself, but also the introduction of a consistent, structured stimulus in an otherwise low-activity environment. From a clinical perspective, preventing decline in this population may be as meaningful as improving performance, given the strong association between functional deterioration and loss of independence, hospitalization, and mortality [65,66]. Importantly, these findings must be interpreted in light of the specific characteristics of institutionalized older adults. This population typically presents advanced age, reduced physiological reserve, high prevalence of chronic disease, sarcopenia, and elevated levels of physical inactivity [60,61,62]. In many nursing homes, structured exercise programmes are minimal or nonexistent, and daily routines are predominantly sedentary, with residents spending a substantial proportion of waking hours inactive [63,64]. Therefore, the magnitude of improvements observed in the intervention groups may reflect the introduction of a consistent, structured stimulus in an otherwise low-activity environment.

From a clinical perspective, improvements in mobility, lower-limb strength, balance-related performance, and walking endurance may be relevant because poorer physical performance in older adults has been associated with subsequent disability, falls, hospitalization, and institutionalization [64,66,67,68]. However, falls, hospitalization, and loss of independence were not directly assessed in the present study; therefore, the observed functional improvements cannot be interpreted as evidence that these adverse outcomes were reduced.

Across several outcomes, the control group showed either no improvement or deterioration over the 12-week study period, including measures of muscular strength, functional mobility, walking endurance, and selected body-composition variables. This pattern should be interpreted in the context of the high prevalence of frailty, sarcopenia, low habitual physical activity, and functional vulnerability commonly observed in institutionalized older adults. However, the present study was not designed to characterize the natural history or expected rate of decline in this population. Therefore, the changes observed in the control group should not be attributed solely to the absence of structured exercise, as unmeasured differences in health status, usual activities, medication use, and institution-level factors may also have contributed.

Taken together, the findings indicate that participation in both face-to-face and videoconference-supervised combined exercise programmes was associated with favourable changes in body composition, muscular strength, flexibility, functional mobility, and aerobic endurance in institutionalized older adults. No statistically significant differences in change were detected between the two supervised delivery formats across most outcomes. However, given the non-randomized design and the absence of a formal equivalence or non-inferiority analysis, these findings should not be interpreted as demonstrating equivalent effectiveness.

Importantly, the similarity in outcomes between FG and VG across most variables suggests that videoconference-based supervised exercise may constitute a feasible alternative to traditional face-to-face delivery. Although the non-randomized controlled study design and sample size warrant cautious interpretation, the present findings indicate that, when supervision and exercise prescription are adequately maintained, remote training may elicit similar physiological and functional adaptations in this population.

### 4.5. Implications for Practice

Taken together, the present findings indicate that both face-to-face and videoconference-supervised combined exercise programs were associated with improvements in body composition, muscular strength, flexibility, functional mobility, and aerobic endurance in institutionalized older adults. The lack of improvement, or deterioration, observed in the control group further supports the interpretation that the changes observed in the intervention groups were related to participation in the exercise programs.

Importantly, the broadly similar patterns of improvement observed in the face-to-face and videoconference groups across most outcomes suggest that videoconference-supervised exercise may represent a viable alternative to traditional face-to-face delivery. However, given the non-randomized controlled study design and the absence of a formal equivalence or non-inferiority analysis, the present findings should not be interpreted as demonstrating statistical equivalence between the two delivery modalities. Nevertheless, the results suggest that, when the exercise content and level of supervision are maintained, remote delivery may produce meaningful physical and functional benefits in institutionalized older adults.

The present findings also have relevant implications for the development and implementation of exercise interventions in older populations. Supervised exercise delivered via videoconference may help expand access to structured physical activity programs, particularly among individuals with mobility limitations or living in settings where regular face-to-face supervision is difficult to provide. This delivery modality may be especially relevant in rural areas, long-term care institutions, and other contexts characterized by geographical, logistical, or resource-related barriers.

The findings should, however, be interpreted in relation to the specific implementation characteristics of the videoconference intervention. The program used a standardized set of exercises selected and adapted to the functional characteristics of institutionalized older adults, together with real-time professional supervision and predefined safety procedures. These elements may have supported appropriate exercise execution, adherence, and participant safety; however, their individual contribution to the observed outcomes was not independently evaluated. Accordingly, the findings should not be generalized to unsupervised remote exercise programs or to interventions using substantially different exercise content, safety procedures, or levels of professional supervision.

The implementation of videoconference-supervised exercise in institutional settings should therefore include appropriately qualified professionals, individualized exercise adaptations, clear safety procedures, and sufficient technological support to enable effective communication and observation of participants. Nevertheless, the present study did not compare different technological or supervision configurations. Consequently, no conclusions can be drawn regarding the superiority of specific camera arrangements or other technological setups. Future studies should directly evaluate which supervision strategies and technological configurations provide the greatest feasibility, safety, and effectiveness for institutionalized older adults.

Exercise programs adapted to different contexts and combining face-to-face, remote, or hybrid delivery modalities may help increase participation among individuals with varying levels of mobility, clinical complexity, and access to services. However, the selection of the delivery format should consider participants’ functional and cognitive abilities, staffing resources, technological infrastructure, and the capacity of each institution to ensure adequate supervision and safety. Integrating these approaches into community services and long-term care institutions may contribute to promoting physical function, autonomy, and healthy aging.

### 4.6. Study Limitations and Future Recommendations

Although the results are promising, it is important to acknowledge certain limitations of this study. The non-randomized controlled study design limits causal inference, and the relatively small sample size may reduce the generalizability of the findings. Additionally, the sample was restricted to a specific geographic region and to elderly people living in residential houses, which further constrains external validity. Although heart rate was monitored in real time to guide exercise intensity, session-level heart-rate data were not systematically recorded; therefore, actual session-level exercise intensity and internal load could not be quantified retrospectively.

Importantly, potential confounding factors were not fully controlled and should be considered when interpreting the findings. These include dietary habits, medication use (e.g., corticosteroids or other drugs that may influence muscle mass, inflammation, or cardiovascular function), baseline levels of physical activity, cognitive status and the presence and severity of comorbidities. Additionally, the age difference observed between groups (with the videoconference group being approximately 4–5 years younger than the face-to-face group) may have influenced adaptive capacity, muscular gains, and cardiorespiratory responses (e.g., VO_2_), thereby potentially affecting the magnitude of training-related improvements.

Medication-related factors warrant additional consideration in this population. Although CNS-active medication burden was incorporated into the sensitivity analyses, residual confounding related to specific drug classes, dosage, treatment duration, drug–drug interactions, and other potentially inappropriate medications cannot be excluded. Polypharmacy and CNS-active medications may contribute to fatigue, sedation, orthostatic hypotension, impaired balance, altered cardiovascular responses, and reduced exercise tolerance, with potential implications for both physical performance and exercise safety [39]. Medication-related risk assessment in vulnerable older adults may therefore benefit from integrating clinical characteristics, functional status, and pharmacological information rather than relying solely on medication counts. Such an integrated approach may facilitate the identification of individuals at greater risk of adverse drug effects and support more individualized exercise monitoring.

Additionally, participants were recruited from five nursing homes, but institution-level clustering was not formally modelled. Given the small number of participating institutions, an institution-level random effect was not estimated; therefore, residual confounding related to differences between nursing homes cannot be excluded.

Future research should explore the effects of longer interventions and examine the efficacy of different intensities and types of remotely delivered exercise programs in other populations. Additionally, the assessment of cost-effectiveness between remote and face-to-face modalities could provide valuable insights for public health policies. It is also recommended that future investigations evaluate strategies to further enhance the benefits of both face-to-face and remote training approaches, as well as examine the effects of hybrid interventions in broader and more diverse populations. Moreover, future studies should consider assessing these exercise interventions across individuals with varying degrees of cognitive impairment or dementia, as well as those without cognitive decline, to better understand how cognitive status may influence responsiveness to physical exercise programs. Future research should also investigate multidisciplinary or hybrid decision-support approaches that integrate clinical characteristics, functional status, medication profiles, and pharmacological risk information to support individualized prescribing and deprescribing decisions in high-risk institutionalized older adults. Such approaches should be prospectively validated in this population before routine clinical implementation.

Future studies should also directly compare different remote supervision strategies and technological configurations, including camera positioning and the number of visual perspectives available to the exercise professional, before specific technological setups can be recommended for routine practice.

## 5. Conclusions

This study provides evidence suggesting that participation in a 12-week supervised combined exercise programme was associated with favourable changes in body composition, muscular strength, flexibility, functional mobility, and aerobic endurance in institutionalized older adults. Both face-to-face and videoconference-supervised interventions were linked to positive adaptations, in contrast to the control group, which displayed no improvement and experienced deterioration in several outcomes.

Importantly, no statistically significant differences in change were detected between the face-to-face and videoconference groups across most outcomes. However, because the study used a non-randomized design and was not designed or powered as an equivalence or non-inferiority trial, these findings should not be interpreted as demonstrating equivalent effectiveness between the two delivery modalities. Nevertheless, videoconference-supervised exercise appears to be a promising delivery option to support physical function in institutionalized older populations, particularly when access to traditional face-to-face interventions is limited. Further randomized studies with longer-term follow-up are warranted to confirm these findings.

## Figures and Tables

**Table 1:** Baseline characteristics of participants by study group.

Baseline			
Variable	Face-to-Face (*n* = 28)	Videoconference (*n* = 32)	Control (*n* = 24)
Age, years (Mean ± SD)	85.75 ± 7.58	80.06 ± 6.83	84.17 ± 7.57
Female (%)	25 (89.3%)	20 (62.5%)	15 (62.5%)
Male (%)	3 (10.7%)	12 (37.5%)	9 (37.5%)
BMI, kg/m^2^ (Mean ± SD)	25.42 ± 5.16	26.98 ± 4.15	22.83 ± 2.45
Illiterate	6	6	7
1–11 years of education	22	26	17
>11 years of education	0	0	0
MMSE Score (Mean ± SD)	20.43 ± 5.94	21.91 ± 4.60	21.13 ± 4.52
Medications with cognitive impact	1.50 ± 1.03	2.19 ± 1.51	1.57 ± 1.21
Medications with physical/balance impact	2.27 ± 1.56	3.00 ± 2.11	2.86 ± 1.77
Medications with combined physical/balance/cognitive impact	2.27 ± 1.56	3.03 ± 2.07	2.86 ± 1.77
CNS-active medications	1.92 ± 1.32	2.77 ± 2.03	2.48 ± 1.57
Prior physical activity	Sedentary/no structured exercise	Sedentary/no structured exercise	Sedentary/no structured exercise

Abbreviations: BMI, body mass index; MMSE, Mini-Mental State Examination; CNS, central nervous system; FG, face-to-face group; VG, videoconference group; CG, control group; SD, standard deviation.

**Table 2:** Physical and functional outcomes across groups and assessment points, with Time, Group, and Time × Group effects.

Variables	Group	Baseline Mean ± SD	6 Weeks Mean ± SD	12 Weeks Mean ± SD	Time	Group	Time × Group
Body mass (kg)	FG	59.99 ± 13.14	60.71 ± 13.01	60.45 ± 12.97	F = 1.277; *p* = 0.280; ηp^2^ = 0.016	F = 2.893; *p* = 0.061; ηp^2^ = 0.067	F = 0.915; *p* = 0.448; ηp^2^ = 0.022
	VG	65.97 ± 9.07	66.09 ± 8.83	65.89 ± 9.59			
	CG	62.72 ± 8.32	62.82 ± 8.50	62.82 ± 8.47			
BMI (kg/m^2^)	FG	25.42 ± 5.16	25.72 ± 5.05	25.62 ± 5.10	F = 1.346; *p* = 0.262; ηp^2^ = 0.016	F = 6.928; *p* = 0.002 *; ηp^2^ = 0.146	F = 0.767; *p* = 0.531; ηp^2^ = 0.019
	VG	26.98 ± 4.15	27.03 ± 4.18	26.94 ± 4.34			
	CG	22.83 ± 2.45	22.87 ± 2.51	22.87 ± 2.49			
Body fat (%)	FG	24.23 ± 4.21	19.15 ± 3.08	18.44 ± 2.91	F = 335.886; *p* < 0.001 *; ηp^2^ = 0.808	F = 7.088; *p* = 0.001 *; ηp^2^ = 0.151	**F = 94.475;** ***p*** < 0.001 *; **ηp^2^ = 0.703**
	VG	25.08 ± 3.22	20.15 ± 2.43	19.60 ± 2.28			
	CG	23.51 ± 3.07	23.62 ± 3.27	23.99 ± 3.20			
Fat-free mass (kg)	FG	44.75 ± 6.39	48.22 ± 6.90	48.51 ± 7.27	F = 112.842; *p* < 0.001 *; ηp^2^ = 0.594	F = 3.988; *p* = 0.022 *; ηp^2^ = 0.094	**F = 29.789;** ***p*** < 0.001 *; **ηp^2^ = 0.436**
	VG	49.25 ± 5.67	52.66 ± 6.28	52.85 ± 6.90			
	CG	47.90 ± 6.04	47.90 ± 6.25	47.67 ± 6.18			
Waist (cm)	FG	100.50 ± 11.66	97.89 ± 11.00	95.30 ± 10.43	F = 67.385; *p* < 0.001 *; ηp^2^ = 0.460	F = 2.338; *p* = 0.103; ηp^2^ = 0.056	**F = 17.846;** ***p*** < 0.001 *; **ηp^2^ = 0.311**
	VG	105.98 ± 7.77	102.44 ± 6.97	100.37 ± 6.50			
	CG	99.52 ± 9.21	100.09 ± 9.22	99.69 ± 9.40			
Hip (cm)	FG	107.39 ± 9.70	104.41 ± 9.73	101.79 ± 10.51	F = 94.209; *p* < 0.001 *; ηp^2^ = 0.547	F = 2.174; *p* = 0.121; ηp^2^ = 0.053	**F = 29.284;** ***p*** < 0.001 *; **ηp^2^ = 0.429**
	VG	105.15 ± 8.32	102.37 ± 8.40	100.74 ± 7.62			
	CG	99.50 ± 4.70	99.89 ± 4.65	99.96 ± 4.66			
Sit-stand test (reps)	FG	9.46 ± 2.69	11.00 ± 2.47	13.05 ± 3.11	F = 57.812; *p* < 0.001 *; ηp^2^ = 0.416	F = 13.711; *p* < 0.001 *; ηp^2^ = 0.253	**F = 27.288;** ***p*** = 0.001 *; **ηp^2^ = 0.403**
	VG	10.56 ± 2.84	12.28 ± 2.72	13.09 ± 2.92			
	CG	8.67 ± 2.73	8.54 ± 2.64	8.00 ± 2.45			
Seated medicine ball throw (m)	FG	1.42 ± 0.35	1.64 ± 0.29	1.84 ± 0.33	F = 72.572; *p* < 0.001 *; ηp^2^ = 0.473	F = 7.635; *p* < 0.001 *; ηp^2^ = 0.159	**F = 37.201;** ***p*** < 0.001 *; **ηp^2^ = 0.479**
	VG	1.64 ± 0.48	1.78 ± 0.48	1.97 ± 0.46			
	CG	1.41 ± 0.44	1.38 ± 0.45	1.31 ± 0.44			
Handgrip test Right (kg)	FG	11.17 ± 3.98	13.56 ± 4.48	16.23 ± 4.72	F = 69.294; *p* < 0.001 *; ηp^2^ = 0.461	F = 8.483; *p* < 0.001 *; ηp^2^ = 0.173	**F = 54.345;** ***p*** < 0.001 *; **ηp^2^ = 0.573**
	VG	15.22 ± 5.32	17.42 ± 5.28	18.77 ± 5.64			
	CG	13.07 ± 4.57	11.99 ± 4.45	11.28 ± 4.41			
Handgrip test Left (kg)	FG	10.76 ± 4.40	13.20 ± 4.16	15.32 ± 4.40	F = 108.614; *p* < 0.001 *; ηp^2^ = 0.573	F = 6.744; *p* = 0.002 *; ηp^2^ = 0.143	**F = 51.007;** ***p*** < 0.001 *; **ηp^2^ = 0.557**
	VG	15.10 ± 5.69	17.43 ± 6.31	18.60 ± 6.40			
	CG	13.16 ± 4.49	12.62 ± 4.30	12.21 ± 4.47			
Arm Curl (reps)	FG	12.00 ± 3.08	14.43 ± 2.96	17.46 ± 3.89	F = 65.260; *p* < 0.001 *; ηp^2^ = 0.446	F = 3.884; *p* = 0.025 *; ηp^2^ = 0.088	**F = 27.700;** ***p*** < 0.001 *; **ηp^2^ = 0.406**
	VG	13.03 ± 3.10	15.13 ± 2.69	16.91 ± 2.52			
	CG	13.25 ± 4.15	12.96 ± 3.63	12.38 ± 3.36			
Back Scratch Test Right (cm)	FG	51.24 ± 13.64	43.18 ± 11.28	35.96 ± 12.38	F = 120.492; *p* < 0.001 *; ηp^2^ = 0.604	F = 9.528; *p* < 0.001 *; ηp^2^ = 0.194	**F = 34.597;** ***p*** < 0.001 *; **ηp^2^ = 0.467**
	VG	54.66 ± 9.71	48.74 ± 9.11	43.29 ± 9.93			
	CG	57.22 ± 13.41	57.22 ± 13.63	57.99 ± 13.65			
Back Scratch Test Left (cm)	FG	54.66 ± 11.62	47.00 ± 11.93	37.39 ± 13.62	F = 71.175; *p* < 0.001 *; ηp^2^ = 0.471	F = 11.404; *p* < 0.001 *; ηp^2^ = 0.222	**F = 30.250;** ***p*** < 0.001 *; **ηp^2^ = 0.431**
	VG	54.06 ± 9.46	48.83 ± 10.01	44.56 ± 11.84			
	CG	60.52 ± 13.57	60.94 ± 13.65	62.48 ± 15.42			
Chair sit-and-reach test Right (cm)	FG	9.86 ± 11.46	5.39 ± 7.86	3.64 ± 6.21	F = 30.972; *p* < 0.001 *; ηp^2^ = 0.277	F = 5.248; *p* = 0.007 *; ηp^2^ = 0.115	**F = 7.100;** ***p*** < 0.001 *; **ηp^2^ = 0.149**
	VG	17.83 ± 15.93	14.84 ± 14.11	12.02 ± 13.40			
	CG	18.65 ± 19.10	18.65 ± 19.20	18.50 ± 18.91			
Chair sit-and-reach test Left (cm)	FG	10.77 ± 11.81	5.43 ± 8.32	3.45 ± 6.26	F = 42.062; *p* < 0.001 *; ηp^2^ = 0.345	F = 7.149; *p* = 0.001 *; ηp^2^ = 0.152	**F = 14.468;** ***p*** < 0.001 *; **ηp^2^ = 0.266**
	VG	13.45 ± 15.82	11.69 ± 14.54	10.55 ± 13.65			
	CG	21.56 ± 18.52	21.40 ± 18.42	21.29 ± 18.51			
8-foot up and go (s)	FG	18.02 ± 8.68	16.02 ± 7.45	14.48 ± 7.07	F = 45.716; *p* < 0.001 *; ηp^2^ = 0.367	F = 5.448; *p* = 0.006 *; ηp^2^ = 0.121	**F = 48.574;** ***p*** < 0.001 *; **ηp^2^ = 0.552**
	VG	19.08 ± 7.29	16.24 ± 6.60	14.34 ± 6.36			
	CG	21.89 ± 10.13	22.77 ± 9.99	24.16 ± 10.48			
6-min walk test (m)	FG	219.32 ± 113.85	232.12 ± 118.55	259.84 ± 126.37	F = 4.918; *p* = 0.018 *; ηp^2^ = 0.061	F = 5.388; *p* = 0.006 *; ηp^2^ = 0.124	**F = 7.664;** ***p*** < 0.001 *; **ηp^2^ = 0.168**
	VG	165.78 ± 71.63	195.09 ± 85.20	234.63 ± 106.78			
	CG	174.58 ± 113.59	137.70 ± 82.82	134.55 ± 85.56			

Abbreviations: % = Percentage, * = Significant differences, BMI = Body Mass Index, CG = Control Group, cm = Centimeters, FG = Face-to-Face Group, kg = Kilogram, M = Meter, Rep = Repetitions, S = Seconds, VG = Videoconference Group. Bold values indicate statistically significant results (*p* < 0.05).

**Table 3:** Within-group changes in physical and functional outcomes across assessment intervals.

Variables	Group	0–6 Mean Differences; *p*	0–12 Mean Differences; *p*	6–12 Mean Differences; *p*
Body fat (%)	FG	**−5.077;** ***p*** < 0.001 *	**−5.791;** ***p*** < 0.001 *	**−0.713;** ***p*** < 0.001 *
	VG	**−4.929;** ***p*** < 0.001 *	**−5.485;** ***p*** < 0.001 *	**−0.556;** ***p*** < 0.001 *
	CG	0.111; *p* = 0.724	0.484; *p* = 0.168	**0.373;** ***p*** = 0.018 *
Fat-free mass (kg)	FG	**3.468;** ***p*** < 0.001 *	**3.760;** ***p*** < 0.001 *	**0.292;** ***p*** **= 0.282**
	VG	**3.410;** ***p*** < 0.001 *	**3.607;** ***p*** < 0.001 *	**0.197;** ***p*** **= 0.418**
	CG	0.009; *p* = 0.976	−0.222; *p* = 0.578	−0.231; *p* = 0.403
Waist (cm)	FG	**−2.611;** ***p*** < 0.001 *	**−5.204;** ***p*** < 0.001 *	**−2.593;** ***p*** < 0.001 *
	VG	**−3.548;** ***p*** < 0.001 *	**−5.613;** ***p*** < 0.001 *	**−2.065;** ***p*** < 0.001 *
	CG	0.021; *p* = 0.965	0.167; *p* = 0.808	0.146; *p* = 0.779
Hip (cm)	FG	**−2.982;** ***p*** < 0.001 *	**−5.607;** ***p*** < 0.001 *	**−2.625;** ***p*** < 0.001 *
	VG	**−2.774;** ***p*** < 0.001 *	**−4.403;** ***p*** < 0.001 *	**−1.629;** ***p*** < 0.001 *
	CG	0.386; *p* = 0.400	0.455; *p* = 0.344	0.068; *p* = 0.860
Sit-stand test (reps)	FG	**1.536;** ***p*** < 0.001 *	**3.571;** ***p*** < 0.001 *	**2.036;** ***p*** < 0.001 *
	VG	**1.719;** ***p*** < 0.001 *	**2.531;** ***p*** < 0.001 *	**0.813;** ***p*** = 0.003 *
	CG	−0.125; *p* = 0.607	−0.667; *p* = 0.080	−0.542; *p* = 0.085
Seated medicine ball throw (m)	FG	**0.221;** ***p*** < 0.001 *	**0.415;** ***p*** < 0.001 *	**0.194;** ***p*** < 0.001 *
	VG	**0.142;** ***p*** < 0.001 *	**0.335;** ***p*** < 0.001 *	**0.192;** ***p*** < 0.001 *
	CG	−0.029; *p* = 0.358	**−0.101;** ***p*** = 0.016 *	**−0.072;** ***p*** = 0.007 *
Handgrip test Right (kg)	FG	**2.398;** ***p*** < 0.001 *	**5.062;** ***p*** < 0.001 *	**2.664;** ***p*** < 0.001 *
	VG	**2.200;** ***p*** < 0.001 *	**3.542;** ***p*** < 0.001 *	**1.342;** ***p*** < 0.001 *
	CG	**−1.085;** ***p*** < 0.001 *	**−1.793;** ***p*** < 0.001 *	**−0.708;** ***p*** = 0.037 *
Handgrip test Left (kg)	FG	**2.442;** ***p*** < 0.001 *	**4.556;** ***p*** < 0.001 *	**2.114;** ***p*** < 0.001 *
	VG	**2.330;** ***p*** < 0.001 *	**3.497;** ***p*** < 0.001 *	**1.167;** ***p*** < 0.001 *
	CG	−0.540; *p* = 0.104	**−0.943;** ***p*** = 0.008 *	−0.403; *p* = 0.052
Arm Curl (reps)	FG	**2.429;** ***p*** < 0.001 *	**5.464;** ***p*** < 0.001 *	**3.036;** ***p*** < 0.001 *
	VG	**2.095;** ***p*** < 0.001 *	**3.875;** ***p*** < 0.001 *	**1.781;** ***p*** < 0.001 *
	CG	−0.292; *p* = 0.445	−0.875; *p* = 0.128	−0.583; *p* = 0.153
Back Scratch Test Right (cm)	FG	**−8.036;** ***p*** < 0.001 *	**−15.250;** ***p*** < 0.001 *	**−7.214;** ***p*** < 0.001 *
	VG	**−5.919;** ***p*** < 0.001 *	**−11.371;** ***p*** < 0.001 *	**−5.452;** ***p*** < 0.001 *
	CG	0.000; *p* = 1.000	0.761; *p* = 0.570	0.761; *p* = 0.390
Back Scratch Test Left (cm)	FG	**−7.661;** ***p*** < 0.001 *	**−17.268;** ***p*** < 0.001 *	**−9.607;** ***p*** < 0.001 *
	VG	**−5.234;** ***p*** < 0.001 *	**−9.500;** ***p*** < 0.001 *	**−4.266;** ***p*** < 0.001 *
	CG	0.413; *p* = 0.646	1.957; *p* = 0.220	1.543; *p* = 0.254
Chair sit-and-reach test Right (cm)	FG	**−4.482;** ***p*** < 0.001 *	**−6.232;** ***p*** < 0.001 *	**−1.750;** ***p*** = 0.004 *
	VG	**−2.984;** ***p*** < 0.001 *	**−5.812;** ***p*** < 0.001 *	**−2.828;** ***p*** < 0.001 *
	CG	0.000; *p* = 1.000	−0.146; *p* = 0.910	−0.146; *p* = 0.819
Chair sit-and-reach test Left (cm)	FG	**−5.339;** ***p*** < 0.001 *	**−7.321;** ***p*** < 0.001 *	**−1.982;** ***p*** < 0.001 *
	VG	**−1.758;** ***p*** = 0.008 *	**−2.903;** ***p*** < 0.001 *	**−1.145;** ***p*** = 0.001 *
	CG	−0.167; *p* = 0.821	−0.271; *p* = 0.773	−0.104; *p* = 0.789
8-foot up and go (s)	FG	**−1.997;** ***p*** < 0.001 *	**−3.543;** ***p*** < 0.001 *	**−1.546;** ***p*** < 0.001 *
	VG	**−2.843;** ***p*** < 0.001 *	**−4.746;** ***p*** < 0.001 *	**−1.903;** ***p*** < 0.001 *
	CG	**0.879;** ***p*** = 0.035 *	**2.263;** ***p*** < 0.001 *	**1.384;** ***p*** < 0.001 *
6-min walk test (m)	FG	12.791; *p* = 0.423	**40.512;** ***p*** = 0.017 *	**27.721;** ***p*** = 0.002 *
	VG	**29.310;** ***p*** = 0.048 *	**68.849;** ***p*** < 0.001 *	**39.545;** ***p*** < 0.001 *
	CG	**−36.874;** ***p*** = 0.036 *	**−40.024;** ***p*** = 0.030 *	−3.150; *p* = 0.734

Abbreviations: % = Percentage, * = Significant differences, BMI = Body Mass Index, CG = Control Group, cm = Centimetres, FG = Face-to-Face Group, Kg = Kilogram, M = Metre, Rep = Repetitions, S = Seconds, VG = Videoconference Group. Bold values indicate statistically significant results (*p* < 0.05).

## Data Availability

The datasets generated and/or analysed during the current study are not publicly available because they contain potentially sensitive information concerning institutionalized older adults but are available from the corresponding author upon reasonable request and subject to applicable ethical and data protection requirements.

## Supplementary Materials

The following supporting information can be downloaded at: https://www.mdpi.com/article/10.3390/healthcare14203539/s1, Table S1: Exercises for the Older People-Organized by Body Region and Progression; Table S2: The analysis between the groups is based on the differences in relation to the baseline value; Table S3: Baseline *p*-values for between-group comparisons of medication-related variable; Table S4: Adjusted sensitivity analysis of physical and functional outcomes across the 12-week intervention.

## Abbreviations

The following abbreviations are used in this manuscript:

6MWT	Six-Minute Walk Test
ANOVA	Analysis of Variance
BIA	Bioelectrical Impedance Analysis
BMI	Body Mass Index
CG	Control Group
CNS	Central Nervous System
FG	Face-to-Face Group
HRmax	Maximum Heart Rate
MMSE	Mini-Mental State Examination
ROM	Range of Motion
SD	Standard Deviation
SPPB	Short Physical Performance Battery
SPSS	Statistical Package for the Social Sciences
