# Supplementary material for: Physical and Functional Effects of Face-to-Face and Remotely Supervised Combined Exercise in Institutionalized Older Adults: A Non-Randomized Controlled Trial
Source: Healthcare (Basel). 2026 Oct 21;14(20):3539. doi: 10.3390/healthcare14203539 (PMC13704628; doi:10.3390/healthcare14203539)
Supplement: Supplementary file 1 [file healthcare-14-03539-s001.zip › healthcare-4560616-Supplementary Material.pdf]

Supplementary Table S1. Exercises for the Older People - Organized by Body Region and Progression

Upper Body (Arms, Shoulders, Chest and Upper Torso)

Progression 1- No Equipment

Front arm raise	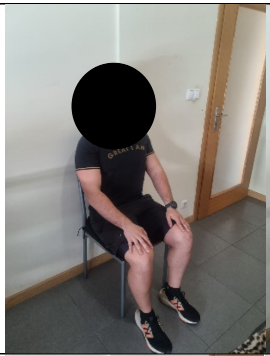 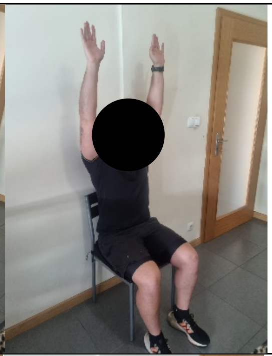
Chest press	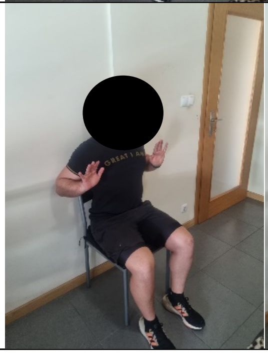 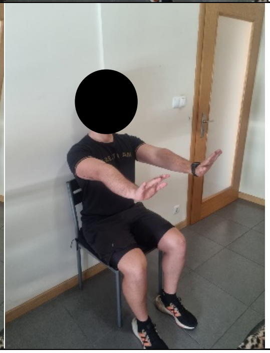

Open and close arms wide	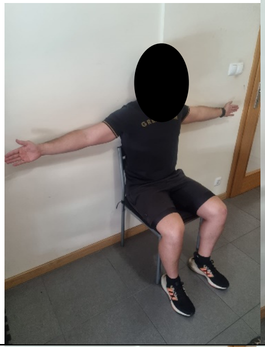 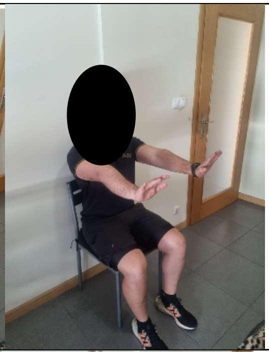
Shoulder press	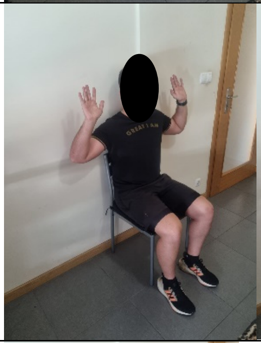 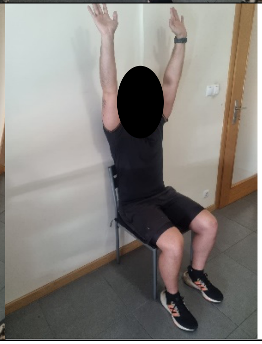
Shoulder shrugs	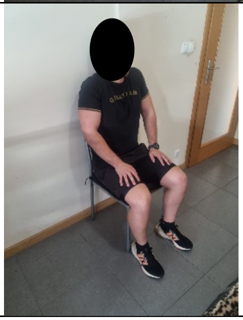

Push one hand against the other	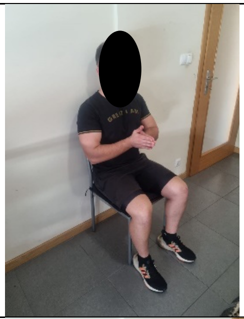
Arms across the chest	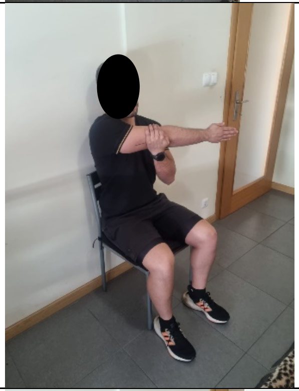
Open and close the hand	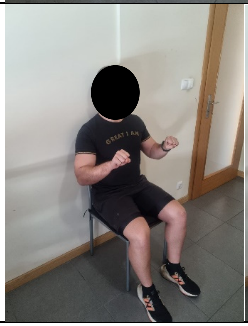 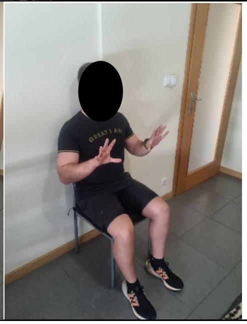

Palm up	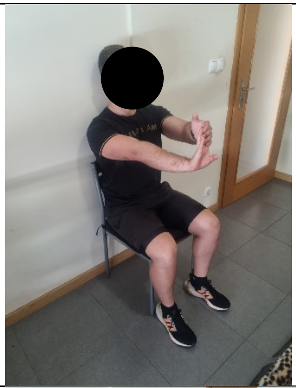
Trunk rotation	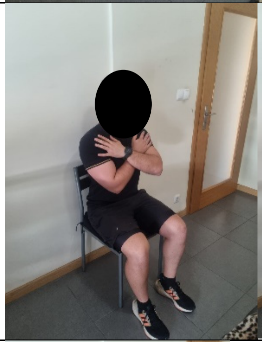 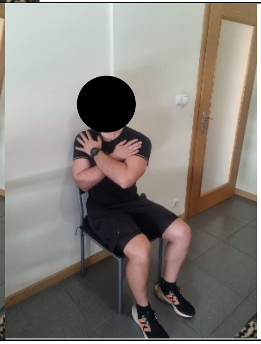
Side bends	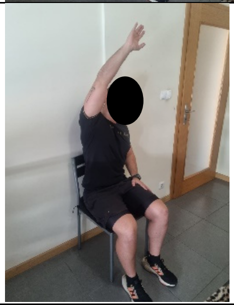

Forward flexion and extension	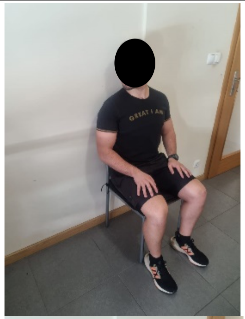 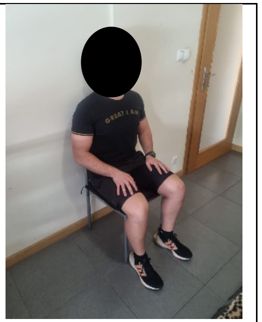 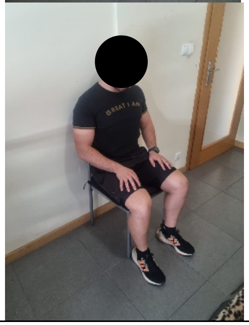

Progression 2 – With Ball

Raise ball above the head	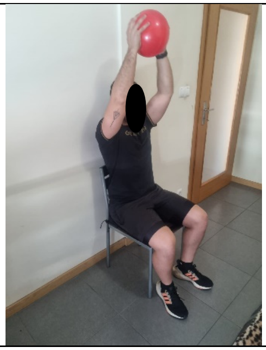

Bring ball behind the head	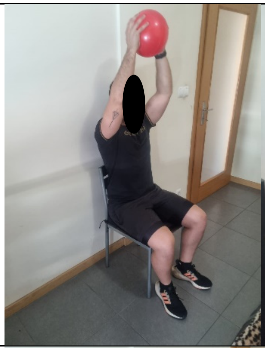 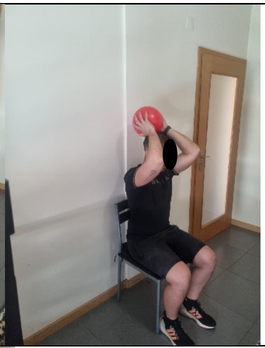
Squeeze ball under the arm	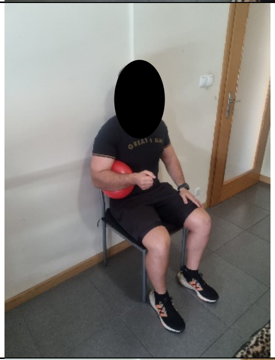
Squeeze ball with hands	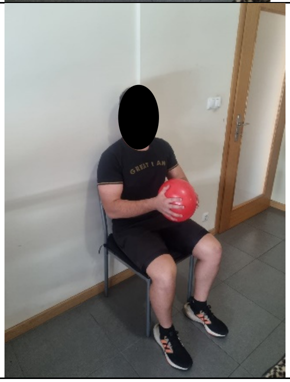

Bring arms forward and squeeze the ball	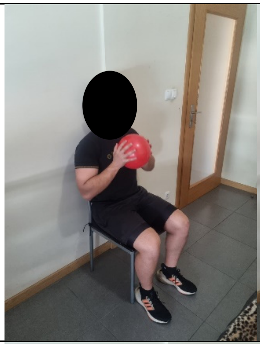 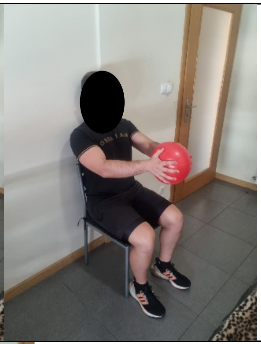
Trunk rotation with ball	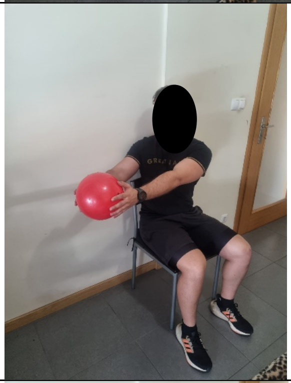
Grab the ball and do not let it drop	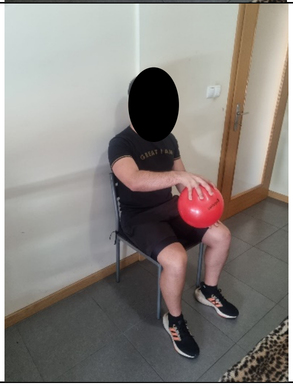

Arm rotations	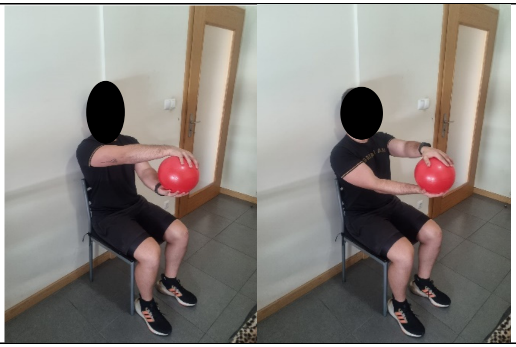

Progression 3 - With Elastic Band

90° arm openers	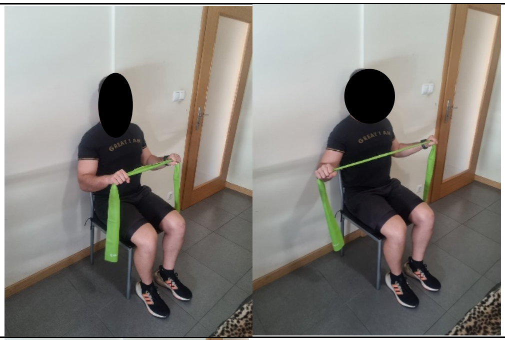
Row with elastic band	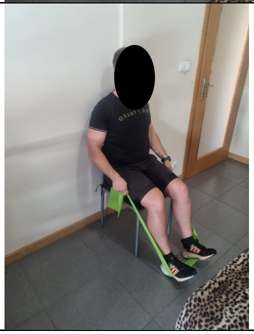

Lateral raise with elastic band	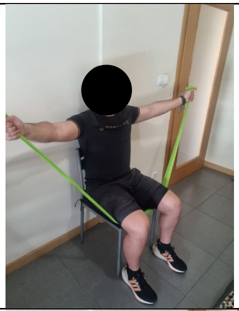
Front raise with elastic band under the leg	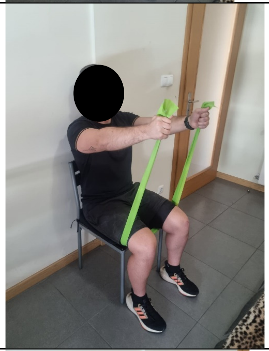
Place one hand on the opposite shoulder and extension the arm	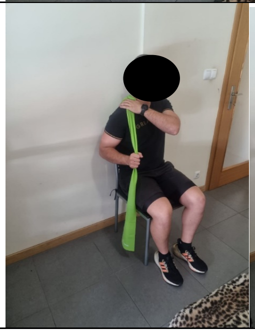 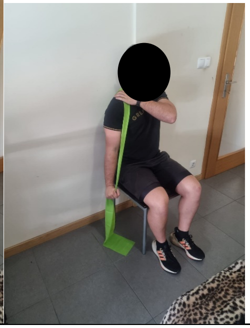

Front raise and Rotation with elastic band	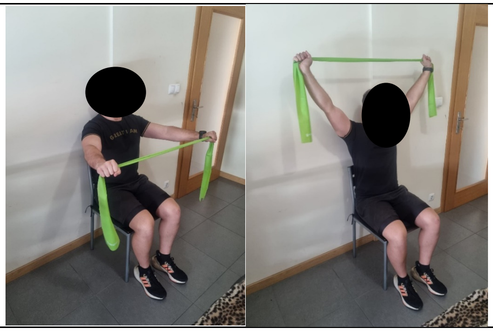

Progression 4 - With Weights

Front raise	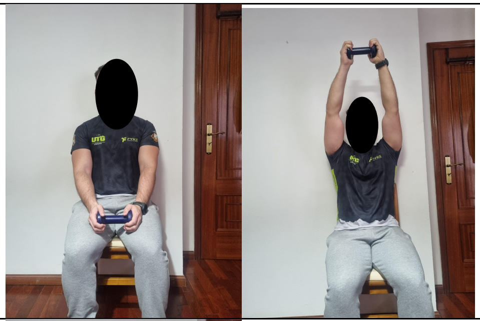
Arm extension to the front	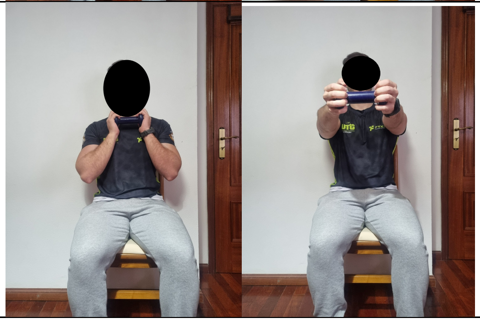

Trunk rotation	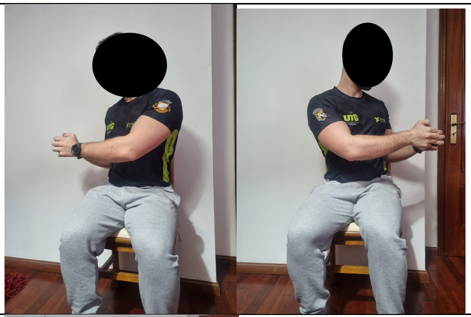
Bicep curl	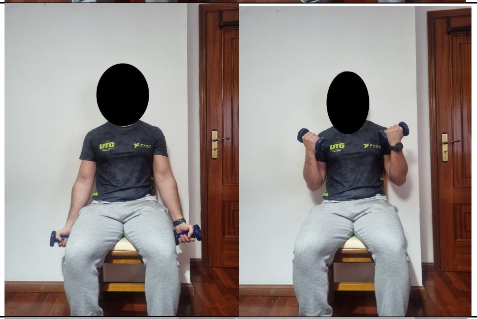
Front rotation	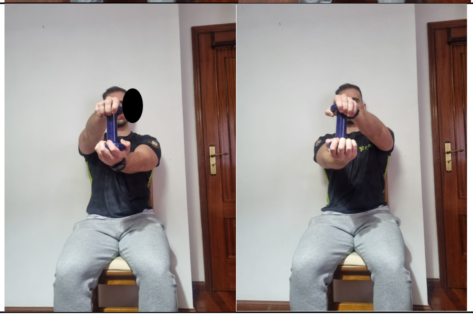

Alternating front raise	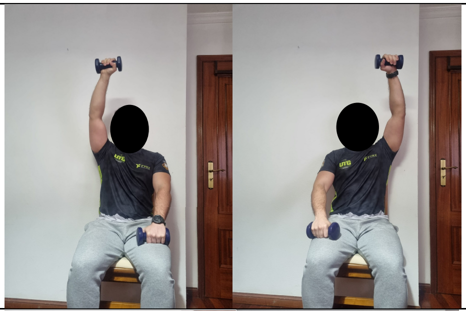
Lateral raise	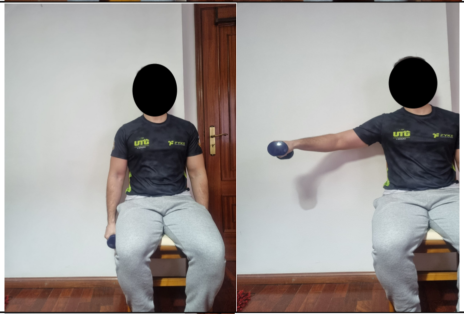
Shoulder shrugs	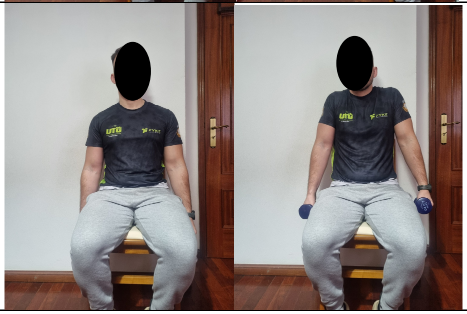

Unilateral shoulder press	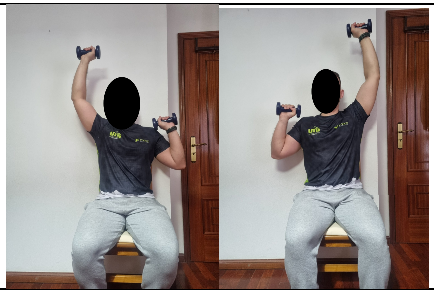

Lower Body (Legs, Knees, Feet and Lower Trunk)

Progression 1 - No Equipment

Foot extension and flexion (Sit upright on a chair with both legs extended together. Point toes away from the body. Then move toes toward the body.)	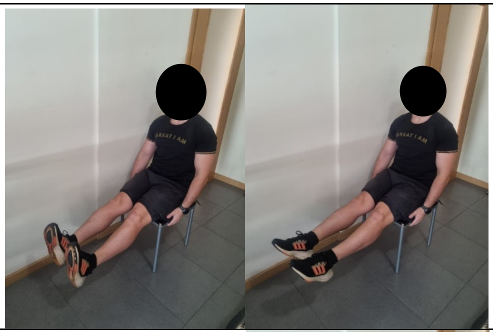
Leg Extension forward (one or both legs)	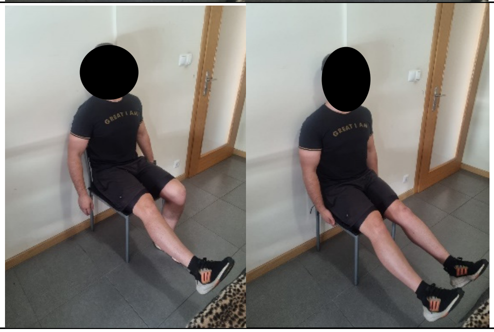

Calf raises	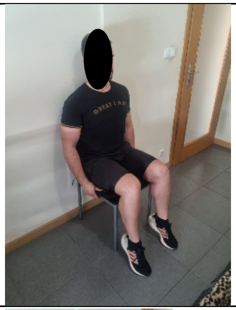
Squats	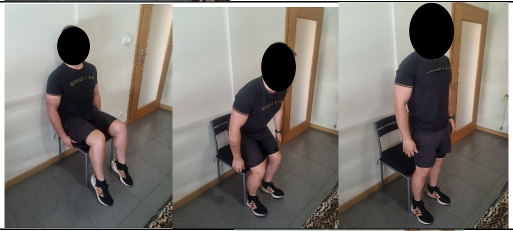
Straight leg abduction	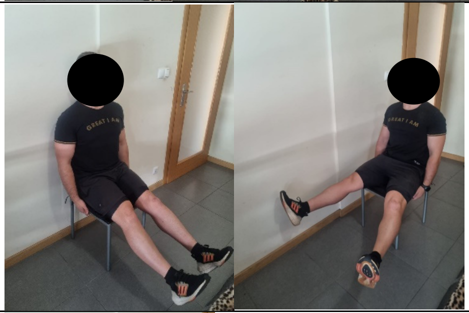
Pull knee	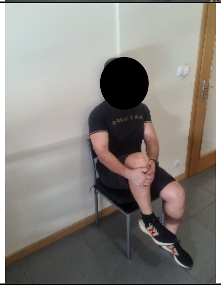

Seated hamstring stretch / Reach foot	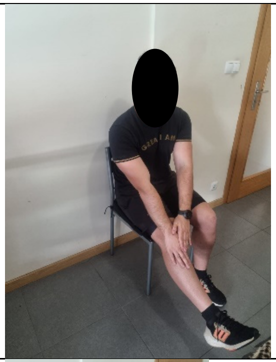
Leg scissors	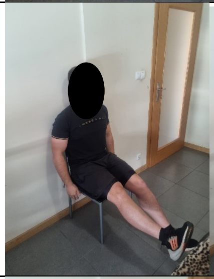 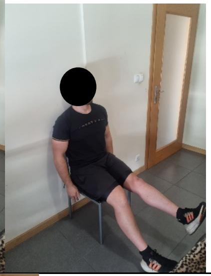
Leg Extension + press	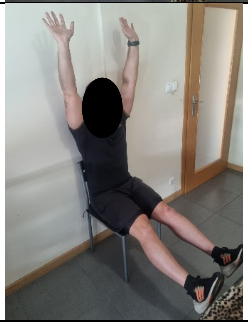

Leg Extension + Chest press	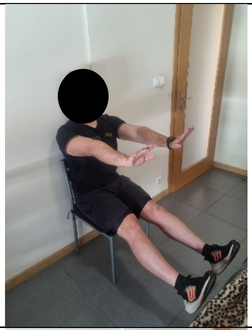
Lift one knee	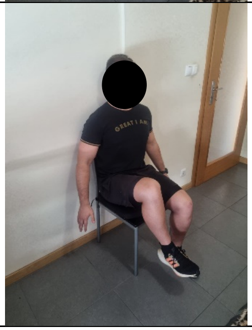

Progression 2 - With Ball

Squat with press	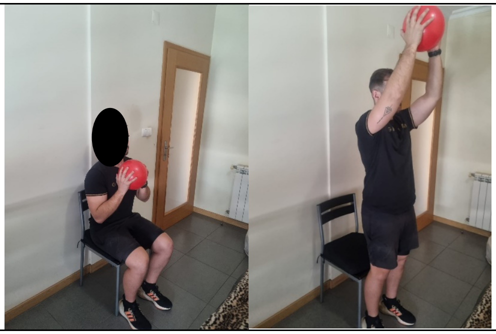

Step on the ball	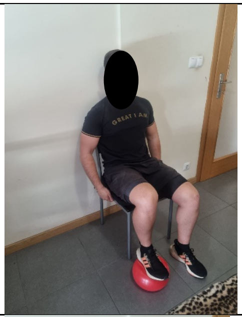
Squeeze ball with the knees	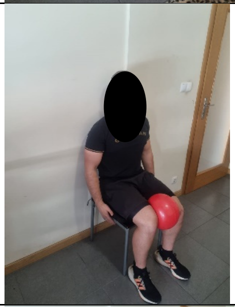
Squeeze ball with the feet and straight legs	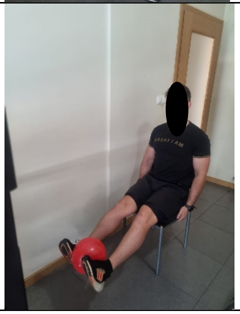

Squeeze ball with one leg behind the other	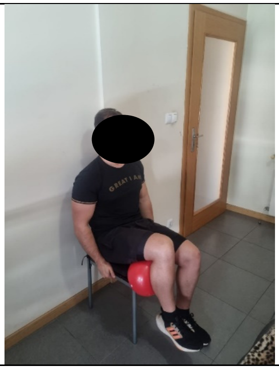

Progression 3- With Elastic Band

Place elastic band around the legs and perform abduction	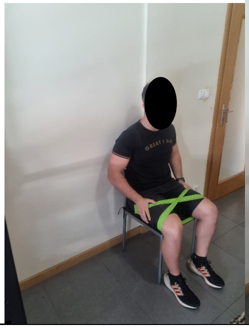 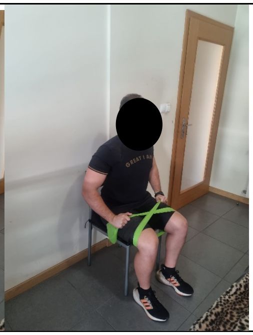

Progression 3- With Weight

Reach foot	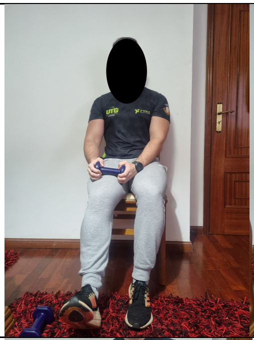 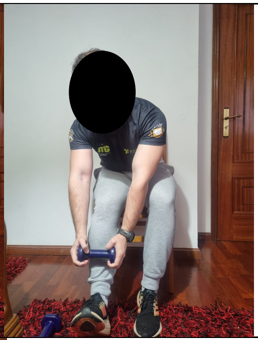
Leg extension	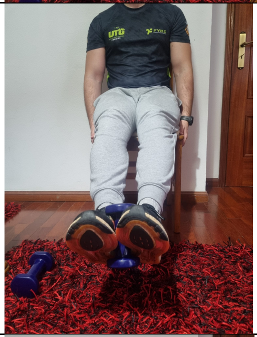
Squat with press	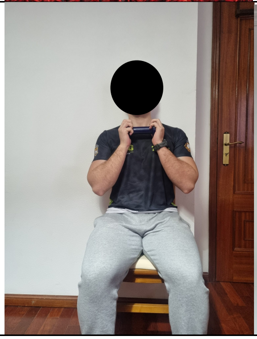 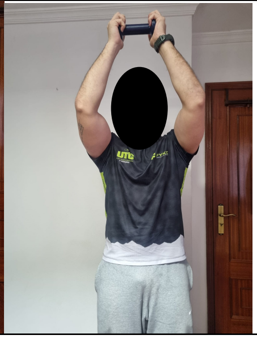

Supplementary Table S2. The analysis between the groups is based on the differences in relation to the baseline value.

**Variables**	Group	Δ_{0-6}<br><br>Mean difference; p	Δ_{0-12}<br><br>Mean difference; p
Body fat (%)	FG Vs VG	-0.312; p=0.884	-0.493; p=1.000
	FG Vs CG	-5.227; p<0.001*	-6.314; p<0.001*
	VG Vs CG	-4.915; p=0.001*	-5.821; p<0.001*
Free fat mass (kg)	FG Vs VG	0.268; p=1.000	0.297; p=1.000
	FG Vs CG	3.518; p<0.001*	4.004; p<0.001*
	VG Vs CG	3.251; p<0.001*	3.707; p<0.001*
Waist (cm)	FG Vs VG	0.937; p=1.000	0.409; p=1.000
	FG Vs CG	-2.632; p<0.001*	-5.370; p<0.001*
	VG Vs CG	-3.569; p<0.001*	-5.780; p<0.001*
Hip (cm)	FG Vs VG	-0.208; p=1.000	-1.204; p=0.572
	FG Vs CG	-3.369; p<0.001*	-6.062; p<0.001*
	VG Vs CG	-3.161; p<0.001*	-4.858; p<0.001*
Sit-stand test (reps)	FG Vs VG	-0.183; p=1.000	1.040; p=1.000
	FG Vs CG	1.661; p<0.001*	4.238; p<0.001*
	VG Vs CG	1.844; p<0.001*	3.198; p<0.001*
Seated medicine ball throw (m)	FG Vs VG	0.079; p=0.717	0.081; p=0.820
	FG Vs CG	0.250; p<0.001*	0.516; p<0.001*
	VG Vs CG	0.172; p<0.001*	0.435; p<0.001*
Handgrip test Right (Kg)	FG Vs VG	0.198; p=1.000	1.520; p=0.336
	FG Vs CG	3.482; p<0.001*	6.855; p<0.001*
	VG Vs CG	3.285; p<0.001*	5.335; p<0.001*
Handgrip test Left (Kg)	FG Vs VG	0.112; p=1.000	1.059; p=0.456
	FG Vs CG	2.982; p<0.001*	5.499; p<0.001*

	VG Vs CG	2.871; p<0.001*	4.440; p<0.001*
Arm Curl (reps)	FG Vs VG	0.335; p=1.000	1.589; p=1.000
	FG Vs CG	2.720; p<0.001*	6.339; p<0.001*
	VG Vs CG	2.385; p<0.001*	4.750; p<0.001*
Back Scratch Test Right (cm)	FG Vs VG	-2.270; p=1.000	-4.266; p=0.794
	FG Vs CG	-8.081; p<0.001*	-16.000; p<0.001*
	VG Vs CG	-5.811; p<0.001*	-11.734; p<0.001*
Back Scratch Test Left (cm)	FG Vs VG	-2.426; p=1.000	-7.768; p=0.065
	FG Vs CG	-8.074; p<0.001*	-19.224; p<0.001*
	VG Vs CG	-5.647; p<0.001*	-11.457; p<0.001*
Chair sit-and-reach test Right (cm)	FG Vs VG	-1.498; p=1.000	-0.420; p=1.000
	FG Vs CG	-4.482; p<0.001*	-6.086; p<0.001*
	VG Vs CG	-2.984; p=0.002*	-5.667; p=0.003*
Chair sit-and-reach test Left (cm)	FG Vs VG	-3.581; p=0.360	-4.418; p=0.271
	FG Vs CG	-5.173; p<0.001*	-7.051; p<0.001*
	VG Vs CG	-1.529; p=0.019*	-2.632; p=0.064
8-foot up and go (s)	FG Vs VG	0.846; p=0.194	1.203; p=0.270
	FG Vs CG	-2.876; p<0.001*	-5.806; p<0.001*
	VG Vs CG	-3.722; p<0.001*	-7.009; p<0.001*
6-minute walk test (m)	FG Vs VG	-3.504; p=0.721	-6.465; p=0.353
	FG Vs CG	39.058; p=0.053	74.883; p=0.012*
	VG Vs CG	42.563; p=0.001*	81.349; p=0.001*

*Abbreviations: % = Percentage, * = Significant differences, BMI = Body Mass Index, CG = Control Group, Cm = Centimetres, ES = Effect Size, FG = Face-to-Face Group, Kg = Kilogram, M = Metre, Rep = Repetitions, S = Seconds,* SpO_2 *= Blood Oxygen Saturation, VG = Videoconference Group*

Supplementary Table S3. Baseline p-values for between-group comparisons of medication-related variables

**Variables**	Group	Total Baseline<br><br>p-value
Number of medications with cognitive impact	FG Vs VG	p=0.180
	FG Vs CG	p=1.000
	VG Vs CG	p=0.469
Number of medications with physical/balance impact	FG Vs VG	p=0.500
	FG Vs CG	p=0.620
	VG Vs CG	p=1.000
Number of medications with physical/balance/cognitive impact	FG Vs VG	p=0.434
	FG Vs CG	p=0.612
	VG Vs CG	p=1.000
Number of active medications in the Central Nervous System	FG Vs VG	p=0.252
	FG Vs CG	p=0.560
	VG Vs CG	p=1.000
≥3 medications active in the Central Nervous System	FG Vs VG	p=0.215
	FG Vs CG	p=0.221
	VG Vs CG	p=1.000

*Abbreviations: * = Significant differences, CG = Control Group, FG = Face-to-Face Group, VG = Videoconference Group*

Supplementary Table S4. Adjusted sensitivity analysis of physical and functional outcomes across the 12-week intervention.

**Variables**	**Group**	**Baseline**<br>**Mean ± SD**	**6 weeks**<br>**Mean ± SD**	**12 week**<br>**Mean ± SD**	**Adjusted Time x Group [a]**	**Adjusted Time x Group [b]**	**Adjusted Time × Group [c]**	**Adjusted Time × Group [d]**
Body mass (kg)	FG	59.99 ± 13.14	60.71 ± 13.01	60.45 ± 12.97	F=1.399; p=0.237; ηp^2=0.036	F = 1.487; p = 0.214; ηp^2 = 0.038	F = 1.241; p = 0.297; ηp^2 = 0.034	F = 1.290; p = 0.279; ηp^2 = 0.036
	VG	65.97 ± 9.07	66.09 ± 8.83	65.89 ± 9.59				
	CG	62.72 ± 8.32	62.82 ± 8.50	62.82 ± 8.47				
BMI (Kg/m2)	FG	25.42 ± 5.16	25.72 ± 5.05	25.62 ± 5.10	F = 0.969; p = 0.426; ηp^2 = 0.025		F = 0.617; p = 0.628; ηp^2 = 0.017	
	VG	26.98 ± 4.15	27.03 ± 4.18	26.94 ± 4.34				
	CG	22.83 ± 2.45	22.87 ± 2.51	22.87 ± 2.49				
Body fat (%)	FG	24.23 ± 4.21	19.15 ± 3.08	18.44 ± 2.91	**F = 76.740; p < 0.001*; ηp^2 = 0.669**	**F = 66.266; p < 0.001*; ηp^2 = 0.639**	**F = 63.170; p < 0.001*; ηp^2 = 0.640**	**F = 54.103; p < 0.001*; ηp^2 = 0.611**
	VG	25.08 ± 3.22	20.15 ± 2.43	19.60 ± 2.28				
	CG	23.51 ± 3.07	23.62 ± 3.27	23.99 ± 3.20				
Fat-free mass (kg)	FG	44.75 ± 6.39	48.22 ± 6.90	48.51 ± 7.27	**F = 23.207; p < 0.001*; ηp^2 = 0.389**	**F = 20.550; p < 0.001*; ηp^2 = 0.363**	**F = 20.740; p < 0.001*; ηp^2 = 0.369**	**F = 19.453; p < 0.001*; ηp^2 = 0.361**
	VG	49.25 ± 5.67	52.66 ± 6.28	52.85 ± 6.90				
	CG	47.90 ± 6.04	47.90 ± 6.25	47.67 ± 6.18				
Waist (cm)	FG	100.50 ± 11.66	97.89 ± 11.00	95.30 ± 10.43	**F = 14.243; p < 0.001*; ηp^2 = 0.275**	**F = 12.211; p < 0.001*; ηp^2 = 0.248**	**F = 11.911; p < 0.001; ηp^2 = 0.251**	**F = 10.025; p < 0.001*; ηp^2 = 0.225**
	VG	105.98 ± 7.77	102.44 ± 6.97	100.37 ± 6.50				
	CG	99.52 ± 9.21	100.09 ± 9.22	99.69 ± 9.40				
Hip (cm)	FG	107.39 ± 9.70	104.41 ± 9.73	101.79 ± 10.51	**F = 23.787; p < 0.001*; ηp^2 = 0.391**	**F = 25.278; p < 0.001*; ηp^2 = 0.409**	**F = 20.469; p < 0.001*; ηp^2 = 0.366**	**F = 22.643; p < 0.001*; ηp^2 = 0.396**
	VG	105.15 ± 8.32	102.37 ± 8.40	100.74 ± 7.62				

	CG	99.50 ± 4.70	99.89 ± 4.65	99.96 ± 4.66				
Sit-stand test (reps)	FG	9.46 ± 2.69	11.00 ± 2.47	13.05 ± 3.11	**F = 18.508; p < 0.001*; ηp² = 0.325**	**F = 18.687; p < 0.001*; ηp² = 0.330**	**F = 15.004; p < 0.001*; ηp² = 0.297**	**F = 14.944; p < 0.001*; ηp² = 0.302**
	VG	10.56 ± 2.84	12.28 ± 2.72	13.09 ± 2.92				
	CG	8.67 ± 2.73	8.54 ± 2.64	8.00 ± 2.45				
Seated medicine ball throw (m)	FG	1.42 ± 0.35	1.64 ± 0.29	1.84 ± 0.33	**F = 26.281; p < 0.001*; ηp² = 0.406**	**F = 26.372; p < 0.001*; ηp² = 0.410**	**F = 20.679; p < 0.001*; ηp² = 0.368**	**F = 20.576; p < 0.001*; ηp² = 0.374**
	VG	1.64 ± 0.48	1.78 ± 0.48	1.97 ± 0.46				
	CG	1.41 ± 0.44	1.38 ± 0.45	1.31 ± 0.44				
Handgrip test Right (Kg)	FG	11.17 ± 3.98	13.56 ± 4.48	16.23 ± 4.72	**F = 56.149; p < 0.001*; ηp² = 0.593**	**F = 51.885; p < 0.001*; ηp² = 0.577**	**F = 46.412; p < 0.001*; ηp² = 0.567**	**F = 42.544; p < 0.001*; ηp² = 0.552**
	VG	15.22 ± 5.32	17.42 ± 5.28	18.77 ± 5.64				
	CG	13.07 ± 4.57	11.99 ± 4.45	11.28 ± 4.41				
Handgrip test Left (Kg)	FG	10.76 ± 4.40	13.20 ± 4.16	15.32 ± 4.40	**F = 50.346; p < 0.001*; ηp² = 0.567**	**F = 45.816; p < 0.001*; ηp² = 0.547**	**F = 38.565; p < 0.001*; ηp² = 0.521**	**F = 36.340; p < 0.001*; ηp² = 0.513**
	VG	15.10 ± 5.69	17.43 ± 6.31	18.60 ± 6.40				
	CG	13.16 ± 4.49	12.62 ± 4.30	12.21 ± 4.47				
Arm Curl (reps)	FG	12.00 ± 3.08	14.43 ± 2.96	17.46 ± 3.89	**F = 17.796; p < 0.001*; ηp² = 0.316**	**F = 19.192; p < 0.001*; ηp² = 0.336**	**F = 15.206; p < 0.001*; ηp² = 0.300**	**F = 16.101; p < 0.001*; ηp² = 0.318**
	VG	13.03 ± 3.10	15.13 ± 2.69	16.91 ± 2.52				
	CG	13.25 ± 4.15	12.96 ± 3.63	12.38 ± 3.36				
Back Scratch Test Right (cm)	FG	51.24 ± 13.64	43.18 ± 11.28	35.96 ± 12.38	**F = 26.028; p < 0.001*; ηp² = 0.410**	**F = 25.838; p < 0.001*; ηp² = 0.411**	**F = 22.589; p < 0.001*; ηp² = 0.389**	**F = 22.539; p < 0.001*; ηp² = 0.395**
	VG	54.66 ± 9.71	48.74 ± 9.11	43.29 ± 9.93				
	CG	57.22 ± 13.41	57.22 ± 13.63	57.99 ± 13.65				
Back Scratch Test Left (cm)	FG	54.66 ± 11.62	47.00 ± 11.93	37.39 ± 13.62	**F = 18.277; p < 0.001*; ηp² = 0.325**	**F = 19.371; p < 0.001*; ηp² = 0.341**	**F = 15.448; p < 0.001*; ηp² = 0.303**	**F = 16.646; p < 0.001*; ηp² = 0.325**
	VG	54.06 ± 9.46	48.83 ± 10.01	44.56 ± 11.84				
	CG	60.52 ± 13.57	60.94 ± 13.65	62.48 ± 15.42				
	FG	9.86 ± 11.46	5.39 ± 7.86	3.64 ± 6.21	**F = 7.954;**	**F = 6.230;**	**F = 5.925;**	**F = 5.303;**

Chair sit-and-reach test Right (cm)	VG	17.83 ± 15.93	14.84 ± 14.11	12.02 ± 13.40	**$p < 0.001$*; $\eta p^2 = 0.171$**	**$p = 0.001$*; $\eta p^2 = 0.141$**	**$p = 0.002$*; $\eta p^2 = 0.143$**	**$p = 0.004$*; $\eta p^2 = 0.133$**
	CG	18.65 ± 19.10	18.65 ± 19.20	18.50 ± 18.91				
Chair sit-and-reach test Left (cm)	FG	10.77 ± 11.81	5.43 ± 8.32	3.45 ± 6.26	**$F = 14.217$; $p < 0.001$*; $\eta p^2 = 0.272$**	**$F = 13.980$; $p < 0.001$*; $\eta p^2 = 0.272$**	**$F = 12.043$; $p < 0.001$*; $\eta p^2 = 0.253$**	**$F = 11.489$; $p < 0.001$*; $\eta p^2 = 0.250$**
	VG	13.45 ± 15.82	11.69 ± 14.54	10.55 ± 13.65				
	CG	21.56 ± 18.52	21.40 ± 18.42	21.29 ± 18.51				
8-foot up and go (s)	FG	18.02 ± 8.68	16.02 ± 7.45	14.48 ± 7.07	**$F = 43.963$; $p < 0.001$*; $\eta p^2 = 0.540$**	**$F = 41.514$; $p < 0.001$*; $\eta p^2 = 0.529$**	**$F = 38.033$; $p < 0.001$*; $\eta p^2 = 0.517$**	**$F = 36.894$; $p < 0.001$*; $\eta p^2 = 0.517$**
	VG	19.08 ± 7.29	16.24 ± 6.60	14.34 ± 6.36				
	CG	21.89 ± 10.13	22.77 ± 9.99	24.16 ± 10.48				
6-minute walk test (m)	FG	219.32 ± 113.85	232.12 ± 118.55	259.84 ± 126.37	**$F = 6.297$; $p < 0.001$*; $\eta p^2 = 0.149$**	**$F = 4.259$; $p = 0.008$*; $\eta p^2 = 0.107$**	**$F = 5.394$; $p = 0.002$*; $\eta p^2 = 0.132$**	**$F = 3.586$; $p = 0.018$*; $\eta p^2 = 0.094$**
	VG	165.78 ± 71.63	195.09 ± 85.20	234.63 ± 106.78				
	CG	174.58 ± 113.59	137.70 ± 82.82	134.55 ± 85.56				

Abbreviations: % = Percentage, * = Significant differences, [a] Adjusted for age and sex, [b] Adjusted for age, sex, and baseline BMI, [c] Adjusted for number of CNS-active medications, [d] Adjusted for age, sex, baseline BMI and number of CNS-active medications, BMI = Body Mass Index, CG = Control Group, Cm = Centimeters, FG = Face-to-Face Group, Kg = Kilogram, M = Meter, Rep = Repetitions, S = Seconds, VG = Videoconference Group. Values correspond to the adjusted Time × Group interaction. Models were adjusted for age and sex.
